# Identification and Validation of a Macrophage Phagocytosis-Related Gene Signature for Prognostic Prediction in Colorectal Cancer (CRC)

**DOI:** 10.3390/cimb47100804

**Published:** 2025-09-29

**Authors:** Xibao Zhao, Binbin Tan, Jinxu Yang, Shanshan Liu

**Affiliations:** Department of Pharmacology, Shenzhen University Medical School, Shenzhen 518055, China

**Keywords:** colorectal cancer, tumor microenvironment, tumor-associated macrophages, phagocytosis, prognosis

## Abstract

Emerging evidence highlights the critical role of phagocytosis-related genes in CRC progression, underscoring the need for novel phagocytosis-based prognostic models to predict clinical outcomes. In this study, a four-gene (SPHK1, VSIG4, FCGR2B and FPR2) signature associated with CRC prognosis was developed using single-sample gene set enrichment analysis (ssGSEA), least absolute shrinkage and selection operator (LASSO) regression, and univariate Cox analysis. Pathway enrichment analysis was conducted on the prognostic genes, along with evaluations of the tumor microenvironment and sensitivity to immunotherapy and chemotherapy across the high- and low-risk groups. Prognostic gene validation was performed via quantitative real-time polymerase chain reaction (qRT-PCR) and immunohistochemistry (IHC) using CRC cDNA and tissue microarrays. High-risk patients showed enhanced responsiveness to immunotherapy, while chemotherapy sensitivity varied across risk subgroups. qRT-PCR results revealed upregulation of SPHK1 and FPR2 in cancer tissues, whereas FCGR2B and VSIG4 were downregulated. IHC assays confirmed increased SPHK1 and FPR2 expression in cancer samples. Single-cell RNA sequencing analysis demonstrated a decrease in SPHK1 and FCGR2B, while VSIG4 and FPR2 progressively increased during macrophage differentiation. These findings provide a potential framework for targeted therapy.

## 1. Introduction

The tumor microenvironment (TME) is a dynamic and complex ecosystem composed not only of cancer cells but also of stromal cells, fibroblasts, endothelial cells, and immune cells [1,2,3]. Macrophages, among the most abundant cell populations in the TME, can constitute up to 50% of the tumor mass in certain solid cancers [4,5]. These cells originate either from bone marrow-derived monocytes or from progenitors in the embryonic yolk sac and liver. Clinical and experimental studies have consistently indicated that tumor-associated macrophages (TAMs) generally exhibit protumoral functions [6]. TAMs interact with other TME cell populations, secrete growth factors such as EGF and PDGF, and release matrix metalloproteinases (MMPs), which promote cancer cell proliferation, tumor invasion, metastasis, and angiogenesis. Furthermore, TAMs contribute to an immunosuppressive TME by inhibiting the activity of cytotoxic T cells and natural killer (NK) cells. This suppression occurs through the expression of immune checkpoint ligands (e.g., PD-L1) and the production of cytokines (e.g., IL10 and TGFβ) and chemokines (e.g., CCL2) [4,5,6,7]. Notably, macrophage actions are mediated by distinct subpopulations within the TME, and their effects may vary depending on the cancer type. The high functional plasticity and heterogeneity of macrophages make them promising targets for novel cancer therapies. These strategies include reducing the recruitment of monocytic progenitors for TAMs, inhibiting the protumoral roles of TAMs, reprogramming protumoral macrophages into antitumoral subtypes, blocking immune checkpoints, disrupting the immunosuppressive TME, and enhancing macrophage-mediated phagocytosis [8].

Phagocytosis is a cellular process in which particles larger than 0.5 μm in diameter are engulfed and digested. Cells involved in phagocytosis, known as phagocytes, include macrophages, neutrophils, monocytes, dendritic cells, and osteoclasts. This process plays critical roles in both physiological and pathological contexts [9,10]. TAM-mediated phagocytosis influences cancer stem cell development and differentiation, tumor invasion, and immune resistance [11]. Moreover, macrophage-related phagocytic factors are essential in regulating TAM polarization, activation, and plasticity, thereby modulating the TME [12,13]. Consequently, phagocytosis holds significant potential for cancer therapy.

Colorectal cancer (CRC) ranks as the third most common malignant tumor and the second leading cause of cancer-related mortality worldwide. In 2022, an estimated 1.9 million new CRC cases and 0.9 million cases occurred globally [14]. The carcinogenesis and progression of CRC involve multiple steps, including genetic mutations, dietary factors, and aging. Risk factors such as smoking, physical inactivity, and obesity further exacerbate the likelihood of CRC development [15]. Current CRC treatments encompass surgical resection, neoadjuvant chemoradiotherapy, postoperative chemoradiotherapy, targeted therapy, and immunotherapy. However, postoperative recurrence, drug resistance, and metastasis continue to pose significant challenges to patient survival following surgery and conventional therapies [16]. Emerging evidence suggests that TAMs contribute to these processes. For instance, chemotherapy with 5-fluorouracil (5-FU) results in the production of diamine putrescine by TAMs in CRC, which in turn induces apoptotic resistance [17]. Additionally, TAMs in CRC recruit a myeloid subtype via CCL1/CCR2 signaling, promoting metastasis [18]. Therefore, targeting TAMs could offer a promising approach to addressing these challenges in CRC treatment. Treatment with a CSF1R antibody, a canonical marker of macrophages, significantly reduced TAM infiltration while increasing the CD8+/CD4+ T cell ratio. However, few patients with CRC respond effectively to anti-CTLA4 and/or anti-PD-1 therapies, potentially due to high PD-1 expression by TAMs, which suppress phagocytosis and cytotoxic immune responses [19]. Notably, a meta-analysis revealed that, unlike in breast, gastric, oral, ovarian, bladder, and thyroid cancers, high TAM infiltration is not associated with poor prognosis in CRC [20]. These findings highlight the functional specificity and complexity of TAMs in CRC. A comprehensive understanding of the roles and regulatory mechanisms of TAMs in CRC is essential for identifying prognostic markers, therapeutic targets, and novel treatment strategies for CRC.

In this study, bioinformatics tools were employed to identify differentially expressed macrophage phagocytosis-related genes in CRC, and a novel prognostic model based on these genes was developed to predict patient survival. The immune microenvironment, immunotherapy sensitivity, and chemotherapy response were evaluated across different risk subgroups. Additionally, intercellular communication and the expression patterns of prognostic genes during macrophage differentiation were explored. These findings provide a theoretical foundation for enhancing prognostic prediction and treatment strategy selection in patients with CRC.

## 2. Materials and Methods

### 2.1. Data Preparation

CRC-related datasets were obtained from the GEO and TCGA databases. The TCGA-CRC dataset consisted of 51 normal and 638 cancer samples. Survival data were available for 606 of these samples, with a median OS follow-up time of 672 days. The GSE17537 dataset (validation set) contained 55 cancer samples with corresponding survival records and a median OS follow-up time of 1500 days. A total of 214 phagocytosis-related genes were identified based on a previously published study [21]. The GSE231559 dataset containing 26 cancer samples was used for scRNA-seq analysis due to its comprehensive cellular coverage and suitability for investigating macrophage-related processes in the CRC microenvironment. Clinical pathological data, including patient age, gender, tumor staging, and treatment plans, as well as the comparison of baseline characteristics between the training and validation datasets, are presented in Supplementary Table S1.

### 2.2. Macrophage Phagocytosis-Related Genes Identification and Enrichment Analysis

Differentially expressed phagocytosis-related genes in the TCGA-CRC dataset were identified using the ‘DEseq2’ package (version 1.30.1) (adjusted *p* < 0.05 and |Log2FC| > 0.5) [22]. Visualization of differentially expressed genes was performed using a volcano plot created with the ‘ggpubr’ package (R package version 0.4.0, https://CRAN.R-project.org/package=ggpubr (accessed on 30 December 2022)) and a hierarchical clustering heatmap created using the ‘pheatmap’ package (R package version 1.0.12, https://CRAN.R-project.org/package=pheatmap (accessed on 30 December 2022)).

Macrophage-specific marker genes were extracted following a previously established method [23]. Based on these macrophage marker genes, the macrophage enrichment scores in the TCGA-CRC cohort were calculated using the ssGSEA algorithm. The Pearson method was then applied to assess the correlation between macrophage enrichment scores and differentially expressed phagocytosis-related genes [24]. Macrophage-related phagocytosis-related genes were selected based on *p* < 0.05 and |cor| > 0.5. Kaplan–Meier (K-M) survival analysis based on overall survival OS and disease-specific survival (DSS) was conducted to explore survival differences between high- and low-expression groups. OS was defined as the time from the initial diagnosis until death from any cause. Patients who were still alive at the end of the study were censored at their last follow-up date. DSS was defined as the time from diagnosis to death due to CRC. Deaths from other causes were censored at the date of last follow-up. Gene Ontology (GO) enrichment analysis was performed using the ‘clusterProfiler’ package (version 3.18.1) [25], and pathway analysis was performed via the Metascape database.

Finally, CRC samples from the TCGA-CRC dataset were categorized according to the median expression of macrophage phagocytosis-related genes. Differences in macrophage enrichment scores between high- and low-expression groups were analyzed using the Wilcoxon test (*p* < 0.05).

### 2.3. Construction and Validation of the Risk Model

The TCGA-CRC dataset (training set) and the GSE17537 dataset (validation set) were used to construct and validate a prognostic risk model. Univariate Cox analysis (survival package [26]) was applied to identify CRC-associated prognostic genes. LASSO regression analysis (glmnet package (version 4.1-4) [27]) was subsequently employed to select significant prognostic genes for the model. Patients were categorized into low- and high-risk groups based on the optimal risk score threshold. The model’s effectiveness was confirmed using receiver operating characteristic (ROC) curves and risk curves. The optimal lambda parameter was selected using lambda.min (which minimizes cross-validation error) rather than lambda.1se to achieve optimal predictive performance given our sample size and data quality.

To robustly evaluate model performance, we employed bootstrap resampling (1000 iterations) to assess the predictive stability of the model at 1-year, 3-year, and 5-year time points. Additionally, we conducted multiple adjustments to the LASSO regression regularization parameters to optimize model performance and confirm the stability and reliability of our findings. We also implemented Elastic Net regression as an alternative approach, using grid search to determine the optimal alpha parameter (ranging from 0 to 1 with 0.1 increments) to evaluate the impact of different regularization strategies on model performance.

### 2.4. Clinical Correlation and Independent Prognostic Analysis

Wilcoxon and Kruskal–Wallis tests were used to analyze differences in clinical features (M, N, T stages, tumor grade, age, and gender) between the two risk groups. K-M curves were used to analyze survival probabilities for subgroups with varying clinicopathological features in the two risk groups. The independent prognostic value of clinical features and risk scores was examined using univariate and multivariate Cox regression models. For independent prognostic analysis, univariate Cox regression was first performed to evaluate the prognostic significance of clinical variables including age, gender, T stage, N stage, M stage, overall stage, tumor grade, and the calculated risk score. Variables achieving statistical significance (*p* < 0.05, HR ≠ 1) in univariate analysis were subsequently entered into multivariate Cox regression analysis to identify independent prognostic factors. The proportional hazards assumption was assessed using the Schoenfeld residuals test for all variables in the multivariate model. A nomogram was constructed incorporating the variables identified as independent prognostic factors using the rms package (R package version 6.3-0, https://CRAN.R-project.org/package=rms (accessed on 30 December 2022)). Furthermore, microsatellite instability (MSI) scores for the TCGA-CRC samples were sourced from public annotations. To evaluate its association with our risk model, we compared MSI scores between the high- and low-risk groups using the Wilcoxon rank-sum test.

### 2.5. Single-Gene Gene Set Enrichment Analysis (GSEA)

Single-gene GSEA using KEGG gene sets identified 13 carcinogenic pathways based on a previously published source [28]. Pathway scores were calculated using the ssGSEA algorithm, and differences in pathway scores between the two risk groups were assessed using the Wilcoxon test. Mantel tests were used to evaluate correlations between risk scores and these pathways, while the Pearson method was used to analyze inter-pathway relationships. Gene set enrichment analysis was performed using the gseKEGG function from the clusterProfiler package with reference to the KEGG database (version 111.0). The background gene set consisted of all human genes (hsa) annotated in KEGG, totaling 18,020 genes. Analysis was carried out using a pre-ranked list of genes based on log_2_ fold changes. Statistical significance was assessed using a dual-threshold approach: a nominal *p*-value < 0.05 served as the primary significance cutoff, and a false discovery rate (FDR) < 0.25 was applied for multiple testing correction. The FDR threshold was chosen in accordance with established GSEA methodology to facilitate the identification of biologically relevant gene sets while controlling the rate of false discoveries [29]. Only enriched pathways satisfying both significance criteria were considered for subsequent downstream analysis.

### 2.6. Tumor Microenvironment (TME) Analysis

Differences in immune score, stroma score, and microenvironment scores between the two risk groups were calculated using the TCGA-CRC dataset. The ssGSEA algorithm was applied to explore the infiltration of 30 cell types (2 stromal cells and 28 immune cells) in the two risk groups. Pearson correlation analysis was used to determine the relationships between risk scores and TME cell types. K-M curves were generated to evaluate the prognostic significance of TME cell types. The combined results of these analyses identified critical TME cells.

### 2.7. Immunotherapy and Chemotherapy Response Analysis

Tumor mutation burden (TMB) was analyzed in both risk groups using the maftools package [30]. Correlations between immune checkpoints and risk scores were then analyzed, followed by a comparative evaluation of immune checkpoint expression in the two risk groups. The TIDE algorithm was used to assess immunotherapy sensitivity. Compounds were sourced from the CTRP and PRISM databases, with duplicates removed. The oncoPredict package [31] was used to calculate drug sensitivity values for each sample. The Spearman method was applied to identify compounds negatively correlated with risk scores, and differences in drug responses between the two risk groups were explored.

### 2.8. Single-Cell RNA Sequencing Analysis

The Seurat package (version 5.0.1) [32] was used to process the scRNA-seq dataset, selecting eligible cells according to the following criteria: (i) cells with fewer than 200 or more than 6000 detected genes were excluded; (ii) genes expressed in fewer than three cells or with unique molecular identifier (UMI) values exceeding 30,000 were removed; and (iii) cells with mitochondrial gene expression exceeding 20% were discarded. The data were normalized, and the 2000 most variable genes were selected using the variance stabilizing transformation (VST) method. Principal component analysis (PCA) was performed to identify principal components (PCs) reflecting expression changes in these genes. Eligible cells were clustered using the FindNeighbors and FindClusters functions from Seurat (version 5.0.1) with a resolution of 0.4 and visualized using uniform manifold approximation and projection (UMAP). Cell cluster annotation was performed by integrating unsupervised clustering results with validation using well-established marker genes from published literature [33] and the CellMarker database (http://117.50.127.228/CellMarker/ (accessed on 30 December 2022)). This approach ensured robust cell type identification through the combination of computational clustering and biological validation. Differential marker genes for each cluster were identified using the FindAllMarkers function (version 5.0.1) with the following parameters: min.pct = 0.25, only.pos = TRUE, and logfc.threshold = 1. To further validate cell type annotations, UMAP visualization of marker gene expression was performed, and functional enrichment analysis was carried out using ReactomeGSA to assess the biological characteristics of distinct cell clusters. Significant differences in cell abundance between cancer and normal samples were detected using the chi-square test (*p* < 0.05), with cells demonstrating statistical significance reclassified as differential subpopulations. Differences in prognostic gene expression between cancer and normal samples across subpopulations were also evaluated (*p* < 0.05), and cells showing significant expression differences in prognostic genes were defined as crucial cells. Crucial cells were identified based on three criteria: (i) significant abundance differences between cancer and normal samples; (ii) differential expression of prognostic genes; and (iii) functional relevance confirmed by cell–cell communication analysis. Cell–cell communication between crucial cells and other cell types was identified using the CellChat database (https://github.com/sqjin/CellChat (accessed on 30 December 2022)). Moreover, pseudotime analysis was performed using the Monocle package (version 2.26.0) [34] to track the differentiation trajectories of crucial cells and detect the expression patterns of prognostic genes along these trajectories.

### 2.9. Quantitative Real-Time PCR (qRT-PCR)

Human CRC cDNA microarrays (Cat# MecDNA-HColA095Su01) containing 15 normal and 80 cancer samples were purchased from Shanghai Outdo Biotech. (Shanghai, China). Real-time PCR was performed using FastStart Universal SYBR Green Master Mix (Rox) (Roche, New York, NY, USA) on a 7500 Real Time PCR System (Applied Biosystems, Carlsbad, CA, USA). The primers used were as follows: SPHK1 Forward: GCTCTGGTGGTCATGTCTGG, SPHK1 Reverse: CACAGCAATAGCGTGCAGT; VSIG4 Forward: GGGGCACCTAACAGTGGAC, VSIG4 Reverse: GTCTGAGCCACGTTGTACCAG; FCGR2B Forward: AGCCAATCCCACTAATCCTGA, FCGR2B Reverse: GGTGCATGAGAAGTGAATAGGTG; FPR2 Forward: TCCTGGTTAACCCAACGAGC, FPR2 Reverse: CCGTGTCATTAGTTGGGGCT. GAPDH Forward: TGTGGGCATCAATGGATTTGG; GAPDH Reverse: ACACCATGTATTCCGGGTCAAT.

### 2.10. Immunohistochemistry (IHC)

Four formalin-fixed, paraffin-embedded human CRC tissue microarray slides (Cat# HLin-Ade147Lym-01) were obtained from Shanghai Outdo Biotech. (Shanghai, China), with each slide containing samples from 49 individual cases. IHC staining was performed as described previously [35]. The primary antibodies used were: rabbit polyclonal anti-SPHK1 (Proteintech, Cat# 10670-1-AP; dilution 1:200, Rosemont, IL, USA), mouse monoclonal anti-VSIG4 (Abcam, Cat# ab252933; dilution 1:100, Cambridge, UK), rabbit polyclonal anti-FCGR2B (Proteintech, Cat# 21541-1-AP; dilution 1:100), and rabbit polyclonal anti-FPR2 (Abcam, Cat# ab203129; dilution 1:3000). Digital slide imaging was performed using a Nikon DS-U3 automated whole-slide scanning system. Protein expression was quantified by a pathologist using the histochemical score (H-score) method, which integrates staining intensity and cellular distribution. All anonymized human colorectal cancer samples were obtained from the National Human Genetic Resources Sharing Service Platform (2005DKA21300). This study was approved by the Ethics Committee of Shenzhen University Medical School (PN-2022-001, 10 January 2022) and Shanghai Outdo Biotech Company (SHYJS-CP-1704010, 7 April 2017 and SHYJS-CP-1407014, 4 July 2014) and was performed in accordance with the Declaration of Helsinki.

### 2.11. Spatial Transcriptomics Validation Analysis

To validate the spatial distribution of macrophages in CRC tissues, spatial transcriptomics data from both CRC patients were analyzed. Data normalization was performed using the SCTransform function in the Seurat package under default settings. Batch effects were removed using the SelectIntegrationFeatures function with nfeatures set to 2000, followed by the PrepSCTIntegration and FindIntegrationAnchors functions. Principal component analysis (PCA) was performed for dimensionality reduction, and the top 20 principal components were selected based on the ElbowPlot results. UMAP clustering was conducted using the FindNeighbors and FindClusters functions with a resolution of 0.3. Multimodal intersection analysis (MIA) was employed to integrate spatial and single-cell transcriptomics data by calculating the hypergeometric enrichment of cell type marker genes across spatial regions, with *p* < 0.05 considered statistically significant.

### 2.12. Statistical Analysis

Statistical analyses and visualizations were conducted using R programming (version 4.0.2) and relevant packages. Kaplan–Meier survival analysis was performed, and statistical significance was evaluated using the Log-rank test. ROC curves were plotted with the timeROC package to compute AUC values along with their 95% confidence intervals. The Wilcoxon rank-sum test and paired *t*-test were applied to compare differences between two groups. For spatial transcriptomics analysis, hypergeometric tests were applied to evaluate cell type enrichment within spatial regions. Differences in cell type proportions between normal and tumor regions were compared using the chi-square test. *p* < 0.05 was considered statistically significant. The *p* values are indicated as follows: * *p* < 0.05, ** *p* < 0.01, *** *p* < 0.001, **** *p* < 0.0001, ns, not significant.

## 3. Results

### 3.1. Identification of Differentially Expressed Macrophage Phagocytosis-Related Genes in CRC

**Figure 1:**
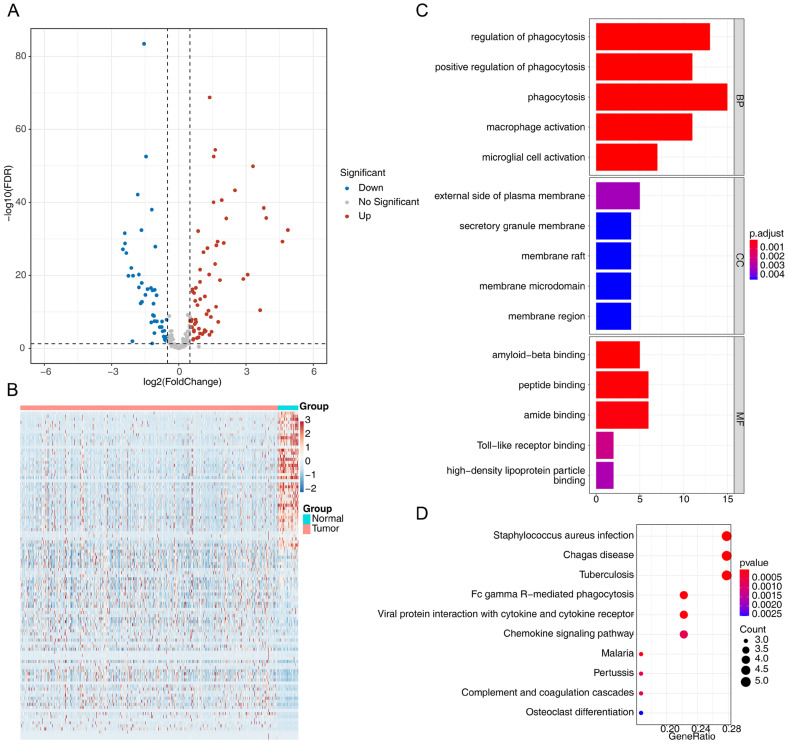
Differentially expressed phagocytosis regulators between CRC tumor and normal tissues in the TCGA-CRC dataset. (**A**,**B**) Volcano plot and heatmap of the differentially expressed phagocytosis regulators. (**C**,**D**) Enriched GO and KEGG pathways related to phagocytosis regulators identified using Single-Gene GSEA.

Using the TCGA-CRC dataset, a total of 107 differentially expressed macrophage-related phagocytosis-related genes were identified, with 62 up-regulated and 45 down-regulated in CRC (Figure 1A,B, Supplementary Table S2). Of these, 20 genes were identified based on the correlation between macrophage enrichment scores and differentially expressed phagocytosis-related genes (Supplementary Figure S1). CRC samples were categorized into high-expression and low-expression groups according to the median expression levels of these genes. Notably, significant differences in macrophage enrichment scores for these 20 phagocytosis-related genes were observed between the two expression subgroups (Supplementary Figure S2).

Enrichment analyses were used to explore the signaling pathways and biological functions associated with these phagocytosis-related genes. The results revealed that processes such as phagocytosis, macrophage activation, phagosome signaling, chemokine signaling, the complement system, cytokine-cytokine receptor interactions, and Fc gamma R-mediated phagocytosis were linked to these genes (Figure 1C,D, Supplementary Table S3).

### 3.2. Development and Validation of a CRC-Related Risk Signature

**Figure 2:**
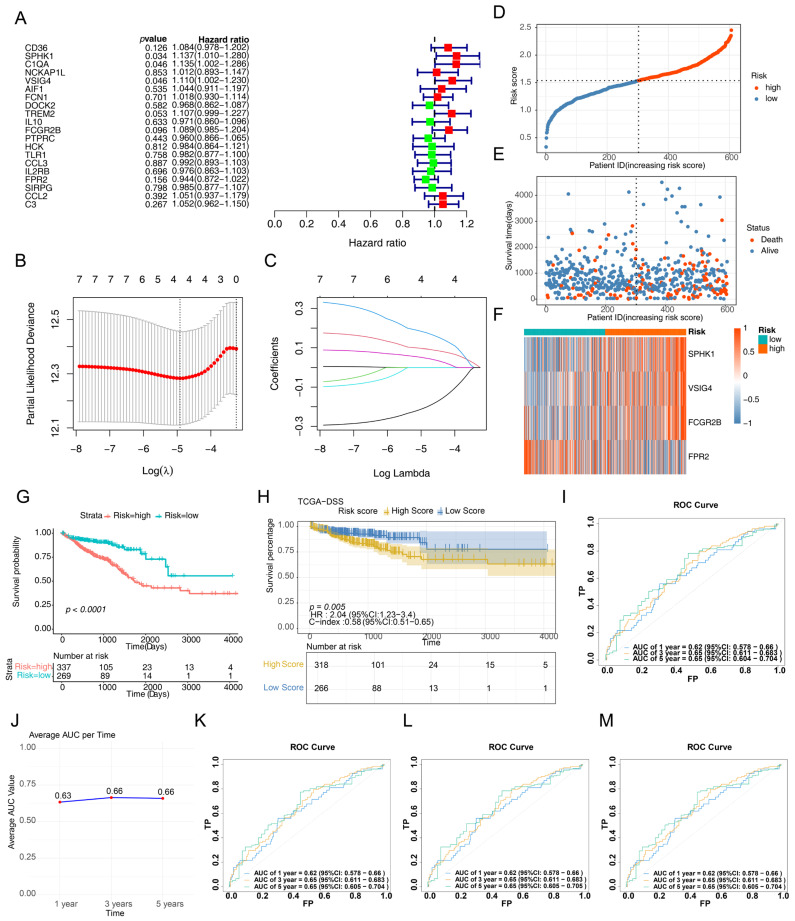
Construction of the prognostic model. (**A**) Forest plot showing the 20 differentially expressed phagocytosis regulatory genes determined by univariate Cox regression analysis. Red indicates that HR is greater than 1, and green indicates that HR is less than 1. (**B**,**C**) LASSO regression analysis identified the four prognostic genes. The dotted line position represents the position with the smallest cross-validation error, and the number of characteristic genes is shown above. The red dots represent the mean values obtained during cross-validation at each λ value. Lines of different colors represent different features. (**D**) Risk score curve classifying CRC patients into high- and low-risk groups based on an optimal threshold. (**E**) Scatter plot showing the survival status of patients in the TCGA-CRC dataset. (**F**) Heatmap presenting the expression of prognostic genes in the high- and low-risk groups. (**G**) OS and (**H**) DSS curves indicating significant differences in survival between the high- and low-risk groups. (**I**) AUC values and 95% confidence intervals are indicated for each time point. The blue curve represents the 1-year ROC curve, the orange curve represents the 3-year ROC curve, and the green curve represents the 5-year ROC curve. (**J**) Bootstrap resampling analysis showing mean AUC values at different time points based on 1000 iterations. (**K**) ROC curves for the LASSO model with lambda = 0.006. (**L**) ROC curves for the LASSO model with lambda = 0.008. (**M**) ROC curves for the Elastic Net model with optimal parameters. The blue curve represents the 1-year ROC curve, the orange curve represents the 3-year ROC curve, and the green curve represents the 5-year ROC curve.

Prognostic genes were screened using univariate Cox analysis for overall survival (OS), and seven genes (CD36, SPHK1, C1QA, VSIG4, TREM2, FCGR2B and FPR2) were identified from the 20 differentially expressed phagocytosis-related genes (*p* < 0.2) (Figure 2A). LASSO regression analysis further narrowed the selection to four prognostic genes (SPHK1, VSIG4, FCGR2B, and FPR2) (Figure 2B,C). A risk score model was then developed to assess patient risk, classifying patients with CRC into high-risk and low-risk groups based on an optimal threshold (Figure 2D,E). Higher risk scores were correlated with shorter survival times. The heatmap results indicated that SPHK1, VSIG4 and FCGR2B were highly expressed in high-risk patients, whereas FPR2 was more highly expressed in low-risk patients (Figure 2F). The OS curves revealed that patients at greater risk had significantly poorer survival (Figure 2G,H). ROC curves demonstrated AUC values for OS of 0.618 (1 year), 0.665 (3 years), and 0.673 (5 years), indicating good performance of this prognostic model (Figure 2I). Similar analyses were performed on the validation dataset (GSE17537), and consistent trends were observed (Supplementary Figure S3). To further validate the stability of the model performance, bootstrap analysis (1000 resampling iterations) demonstrated that the four-gene model achieved mean AUC values greater than 0.6 for 1-year, 3-year, and 5-year predictions, confirming the model’s predictive capability (Figure 2J). Regularization parameter optimization revealed that LASSO models using lambda = 0.006 and lambda = 0.008 showed predictive performance comparable to the original model (lambda.min = 0.00729), indicating robustness to parameter selection (Figure 2K,L). Elastic net analysis identified the optimal alpha value as 1 with lambda.min = 0.006049287 (Figure 2M), yielding predictive performance similar to that of the LASSO model, which further supports the effectiveness of our feature selection strategy.

### 3.3. Correlation Between Risk Scores and Clinicopathological Features

**Figure 3:**
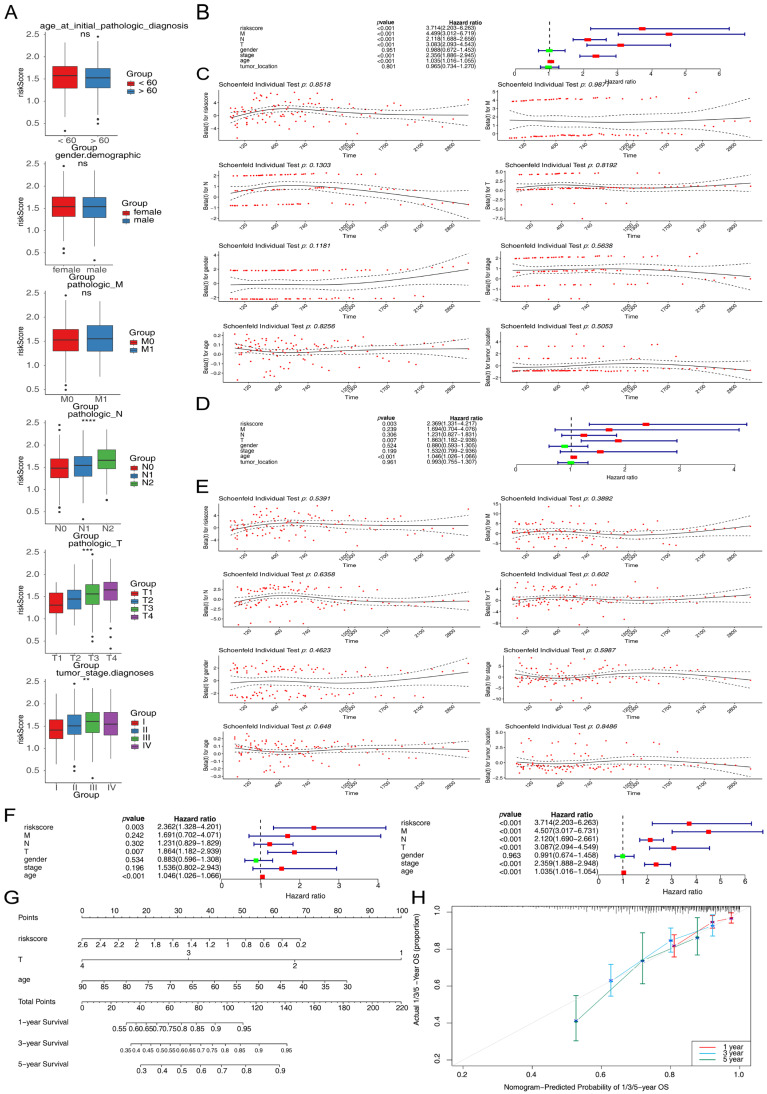
Independent prognostic analysis in the TCGA-CRC dataset. (**A**) Analysis of the correlation between risk score and clinicopathological characteristics. ** *p* < 0.01, *** *p* < 0.001, **** *p* < 0.0001, ns, not significant. (**B**,**C**) Comprehensive univariate Cox regression analysis showed that risk score, age, stage, T stage, M stage, and N stage were significantly associated with CRC patient survival (*p* < 0.05), with all variables satisfying the proportional hazards assumption (*p* > 0.05 in the Schoenfeld residuals test). The dotted line in the middle of the (**B**) is the invalid line, that is, HR = 1, indicating that there is no statistical association between the studied factor and the outcome. If the horizontal line falls to the left of the invalid vertical line, it can be considered that the studied factor is conducive to the occurrence of the outcome. If the horizontal line falls to the right of the invalid line, it can be considered that the studied factor is unfavorable for the occurrence of the outcome. Among them, red indicates that HR is greater than 1, and green indicates that HR is less than 1. The red dots in the (**C**) represent the Schoenfeld residuals calculated at each event occurrence time point. The solid line is a smooth fitting curve for the red residual points. The dotted line represents the confidence interval of the smooth-fitted curve. (**D**,**E**) In multivariate analysis adjusting for age, gender, stage, T stage, M stage, and N stage, risk scores, age, and T stage were identified as independent prognostic factors for CRC (*p* < 0.05), with the proportional hazards assumption maintained for all variables. (**F**) Univariate and multivariate Cox regression analyses of the independent prognostic value of the risk scores and clinicopathological characteristics. (**G**) Nomogram predicting 1-year, 3-year and 5-year survival of CRC patients based on risk score, age, and T stage. (**H**) Calibration curve of the nomogram. The *X*-axis represents the probability predicted by the nomogram, and the *Y*-axis represents the actual probability. Different colors represent different years. Deviation correction is performed through Bootstrapping (1000 repetitions) to indicate the observed performance of the nomogram.

To analyze the relationships between clinical characteristics and risk scores in CRC patients, differences in risk scores across various clinical features were analyzed. Significant variations in risk scores were observed among different tumor grades, N stages, and T stages (Figure 3A). K-M curves indicated lower survival rates for the high-risk group in M0 and T4 stages (Supplementary Figure S4). Comprehensive univariate Cox regression analysis demonstrated that risk score, age, stage, T stage, M stage, and N stage were significantly associated with CRC patient survival (*p* < 0.05), with all variables satisfying the proportional hazards assumption (*p* > 0.05 in Schoenfeld residual test) (Figure 3B,C). In the multivariate analysis adjusted for age, gender, stage, T stage, M stage, and N stage, risk score, age, and T stage were identified as independent prognostic factors for CRC (*p* < 0.05), with the proportional hazards assumption maintained for all variables (Figure 3D,E). The comprehensive results of the univariate and multivariate Cox regression analyses are summarized in Figure 3F. A nomogram model incorporating T stage, age, and risk score was developed to predict patient survival at different time points (Figure 3G), demonstrating reliable predictive performance for CRC patients (Figure 3H). The selection of these covariates was based on established clinical relevance, while tumor location was excluded due to substantial missing data in our cohort. The relationship between the prognostic signature and microsatellite instability (MSI) status was also evaluated. MSI scores did not differ significantly between the high- and low-risk subgroups (Supplementary Figure S5), demonstrating that the signature provides prognostic information independent of MSI status.

### 3.4. Functional Enrichment of the Prognostic Genes

**Figure 4:**
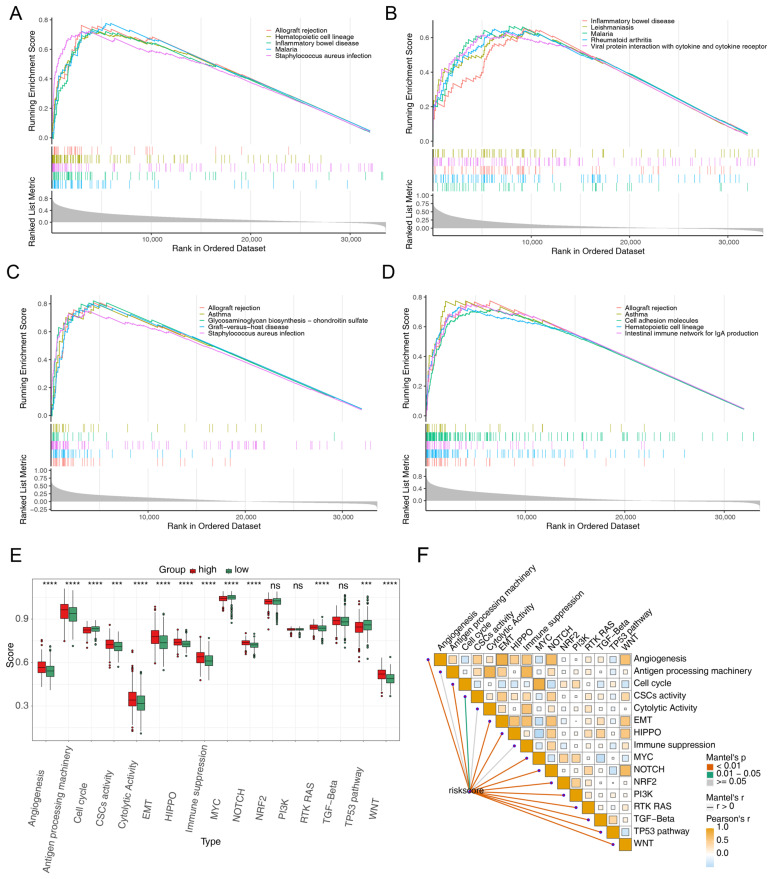
Functional enrichment of the four prognostic genes. (**A**–**D**) Gene set enrichment analysis of (**A**) FCGR2B, (**B**) FPR2, (**C**) SPHK1 and (**D**) VSIG4. (**E**) Comparison of carcinogenic pathway scores between the high- and low-risk groups. *** *p* < 0.001, **** *p* < 0.0001, ns, not significant. (**F**) Correlation analysis between carcinogenic pathways and risk scores.

To investigate the signaling pathways and biological functions associated with the prognostic genes, single-gene GSEA was performed. The results illustrated that Th17 cell differentiation, type I diabetes mellitus, osteoclast differentiation, the Toll-like receptor signaling pathway, Fc gamma R-mediated phagocytosis, and complement and coagulation cascades were related to the four prognostic genes (Figure 4A–D). Significant differences in the scores of most carcinogenic pathways between high- and low-risk groups were observed (Figure 4E). Additionally, risk scores showed a positive correlation with the scores of most carcinogenic pathways (Figure 4F). These results suggest that the four prognostic genes are involved in CRC carcinogenesis.

### 3.5. Different Immune Infiltrates in the Two Risk Groups

**Figure 5:**
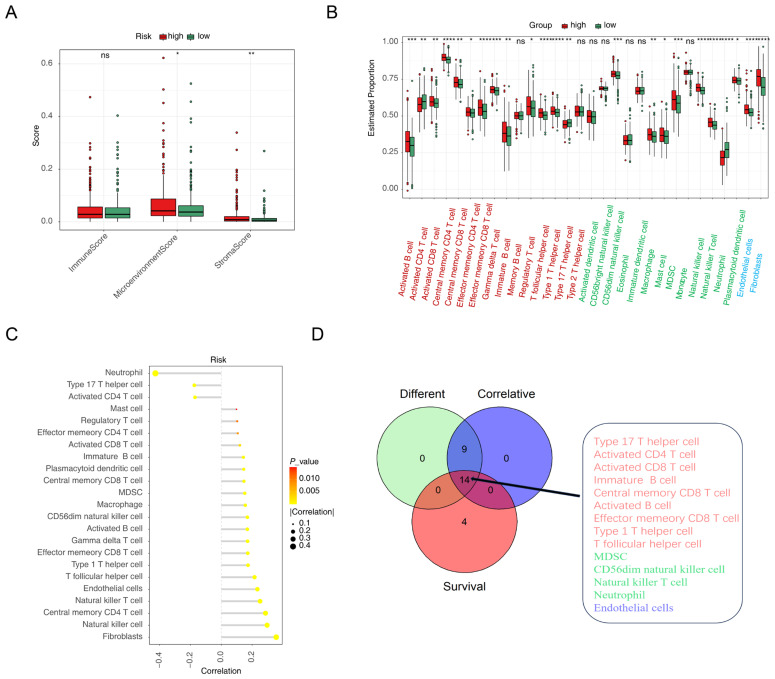
Immune microenvironment landscape in the two risk groups. (**A**) Distribution of the immune score, matrix score, and microenvironment score between the high- and low-risk groups. * *p* < 0.05, ** *p* < 0.01, ns, not significant. (**B**) Differences in immune cell infiltration between the high- and low-risk groups. * *p* < 0.05, ** *p* < 0.01, *** *p* < 0.001, **** *p* < 0.0001, ns, not significant. (**C**) Correlations between risk scores and 23 types of TME cells. (**D**) Venn diagram illustrating the intersection of differential cells, significantly related cells, and cells with prognostic potential. Red: adaptive immune cells. Green: innate immune cells. Blue: fibroblasts and endothelial cells.

TME analysis was conducted to analyze the difference in immune infiltration between the high- and low-risk subgroups. The results revealed a significant difference in the stroma score between the two subgroups (Figure 5A). Immune infiltration analysis showed 23 types of immune cells exhibiting different infiltration patterns between the two risk groups (Figure 5B). We further analyzed the correlation between the risk score and 30 TME cell types, identifying 23 TME cells that significantly correlated with the risk score (Figure 5C). K-M curves indicated that 18 types of TME cells had potential prognostic value for CRC (Supplementary Figure S6). By intersecting these findings, we identified 14 key TME cell types crucial for CRC, including activated B cells, type 1 T helper cells, central memory CD8 T cells, MDSCs, effector memory CD8 T cells, neutrophils, activated CD4 T cells, T follicular helper cells, activated CD8 T cells, CD56dim NK cells, NK T cells, immature B cells, endothelial cells and type 17 T helper cells (Figure 5D).

### 3.6. Different Responses to Immunotherapy and Chemotherapy in the Two Risk Groups

**Figure 6:**
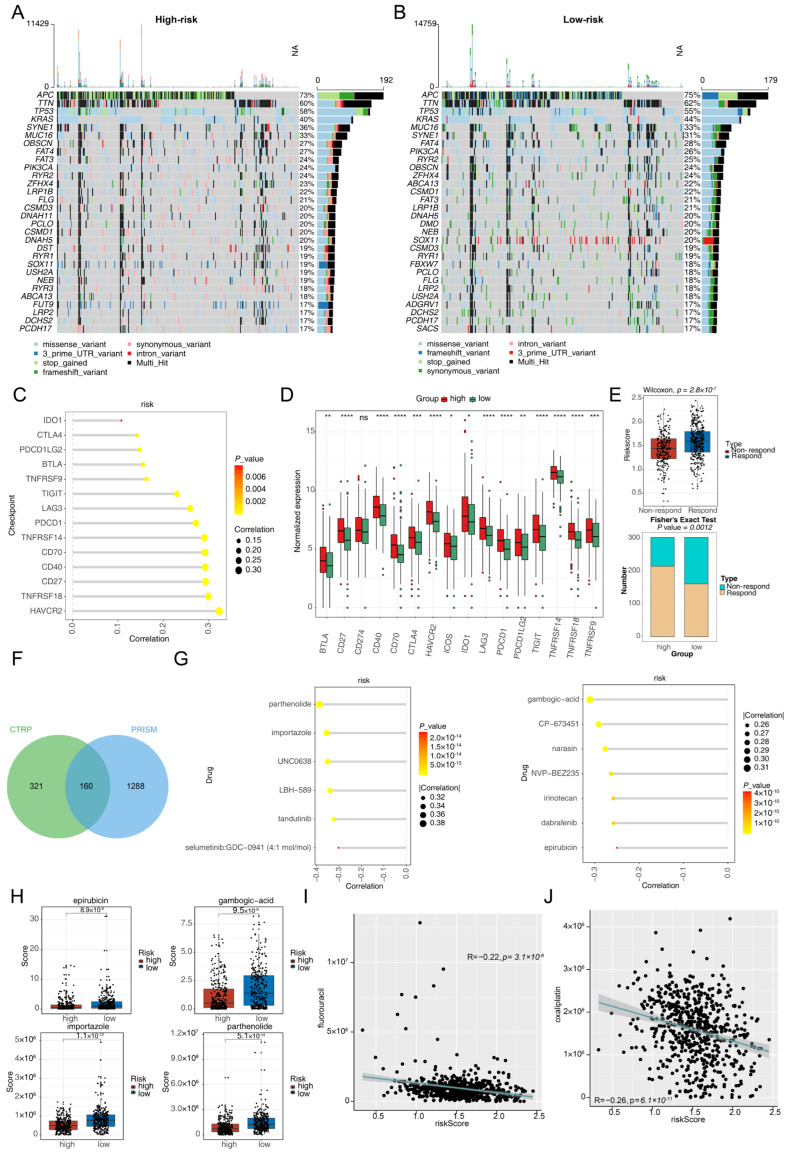
Differences in the response to immunotherapy and chemotherapy between the two risk groups. (**A**,**B**) The top 30 genes with the highest mutation frequencies in (**A**) the high-risk and (**B**) the low-risk groups. (**C**) Correlation analysis of risk scores and immune checkpoints. (**D**) Comparison of immune checkpoint expression between the high- and low-risk groups. * *p* < 0.05, ** *p* < 0.01, *** *p* < 0.001, **** *p* < 0.0001, ns, not significant. (**E**) Comparison of immunotherapy responses between high- and low-risk patients. Upper: risk score distribution in patients who responded to or did not respond to immunotherapy; lower: proportion of responders in the high and low-risk groups. (**F**) Venn diagram of intersecting compounds from the CTRP and PRISM datasets. (**G**) Correlation analysis between risk scores and chemotherapy sensitivity. (**H**) Comparison of sensitivity to four compounds between the high- and low-risk groups. (**I**,**J**) Correlation analysis between risk scores and IC_50_ values of standard CRC chemotherapy drugs 5-fluorouracil (**I**) and oxaliplatin (**J**).

TMB analysis of the two risk groups revealed genes with more than 30 mutations in each group (Figure 6A,B). Additionally, the correlation between risk scores and immune checkpoints was examined, which revealed a significant positive correlation (Figure 6C). In the high-risk group, most immune checkpoints presented elevated expression levels (Figure 6D). Furthermore, a higher number of patients in the high-risk group responded to immunotherapy (Figure 6E), illustrating better immunotherapy responses in high-risk patients.

Chemotherapy sensitivity was assessed using 1770 compounds from the CTRP and PRISM databases (Figure 6F). The results showed that 23 compounds from CTRP and 25 compounds from PRISM were associated with the high-risk group. Subsequent correlation analysis identified 9 compounds (parthenolide, BRD-K71935468, importazole, UNC0638, LBH-589, Ko-143, PLX-4720, tandutinib, and selumetinib: GDC-0941) from CTRP and 10 compounds (gambogic-acid, CP-673451, ponatinib, narasin, norethindrone-acetate, nintedanib, NVP-BEZ235, irinotecan, dabrafenib, and epirubicin) from PRISM significantly negatively correlated with risk scores (Figure 6G). The analysis also revealed significant differences in the sensitivity of the two risk subgroups to four chemotherapeutic agents: parthenolide, importazole, gambogic-acid, and epirubicin (Figure 6H). Additionally, risk scores showed significant negative correlations with IC_50_ values of standard CRC chemotherapy drugs including 5-fluorouracil and oxaliplatin (Figure 6I,J). Consistent with this finding, knockdown of SPHK1 expression using a specific shRNA significantly enhanced cellular sensitivity to 5-fluorouracil in CRC SW480 cells (Supplementary Figure S7).

### 3.7. The Prognostic Genes Functioned Through Being Expressed in Macrophages

**Figure 7:**
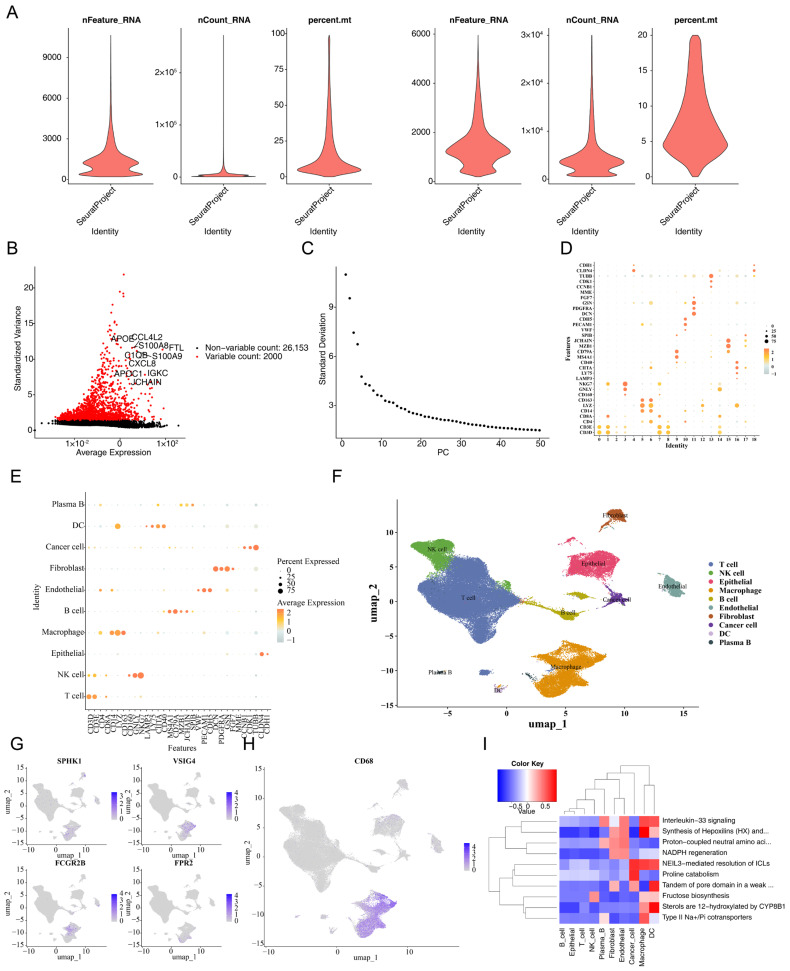
Data processing and cell type identification. (**A**) Comparison of single-cell quality control metrics before and after filtering. (**B**) Screening of highly variable genes. (**C**) Principal component analysis graph. (**D**) Expression of marker genes in 19 clusters. (**E**) Expression of marker genes in 10 annotated cell types. (**F**) Annotation diagram of cell clusters based on the expression of cell-type-specific markers. (**G**) UMAP plots showing the expression distribution of prognostic genes across different cell types. (**H**) UMAP plot showing CD68 expression distribution in macrophage clusters. (**I**) Heatmap of functional enrichment analysis showing pathway activity scores across different cell types.

The expression of the four prognostic genes in macrophages was further analyzed using single-cell RNA sequencing. After filtering out ineligible cells and genes, a total of 86,024 cells and 28,153 genes were retained (Figure 7A), from which the 2000 most variable genes were selected (Figure 7B). The top 30 PCs were used to divide these cells into 19 different clusters (Figure 7C,D and Supplementary Figure S8). Using markers from published literature, the 19 clusters were annotated as 10 cell types: LYZ was predominantly expressed in dendritic cells and macrophages, while NKG7 was mainly found in NK cells (Figure 7E,F). To further validate cell type annotation accuracy, UMAP visualization of marker gene expression was performed. The prognostic genes (SPHK1, VSIG4, FCGR2B, and FPR2) showed more significant expression in macrophages compared to other cell types. Particularly, the macrophage marker gene CD68 demonstrated distinctly high expression in the macrophage cluster, confirming the accuracy of our cell type annotation (Figure 7G,H). Functional enrichment analysis using ReactomeGSA identified 1711 enriched pathways across different cell clusters, with the top enriched pathways including Interleukin-33 signaling, synthesis of Hepoxilins and Trioxilins, proline catabolism, NADPH regeneration, and proton-coupled neutral amino acid transporters (Figure 7I).

**Figure 8:**
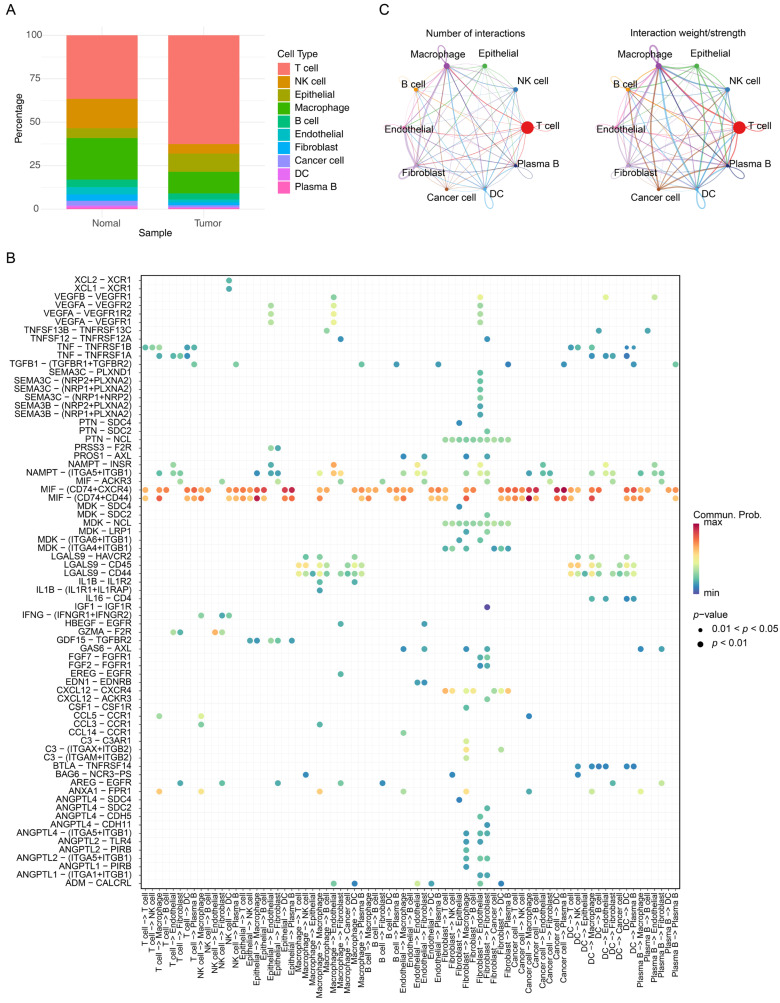
Analysis of cell–cell communication among the 10 annotated cell types. (**A**) Proportions of the 10 annotated cell types between normal and CRC tumor samples. (**B**) Dot plots illustrating specific ligand-receptor interactions between macrophages and other cell types. (**C**) Network diagram showing the quantity (**left**) and strength (**right**) of cell–cell communication among the 10 annotated cell types.

**Figure 9:**
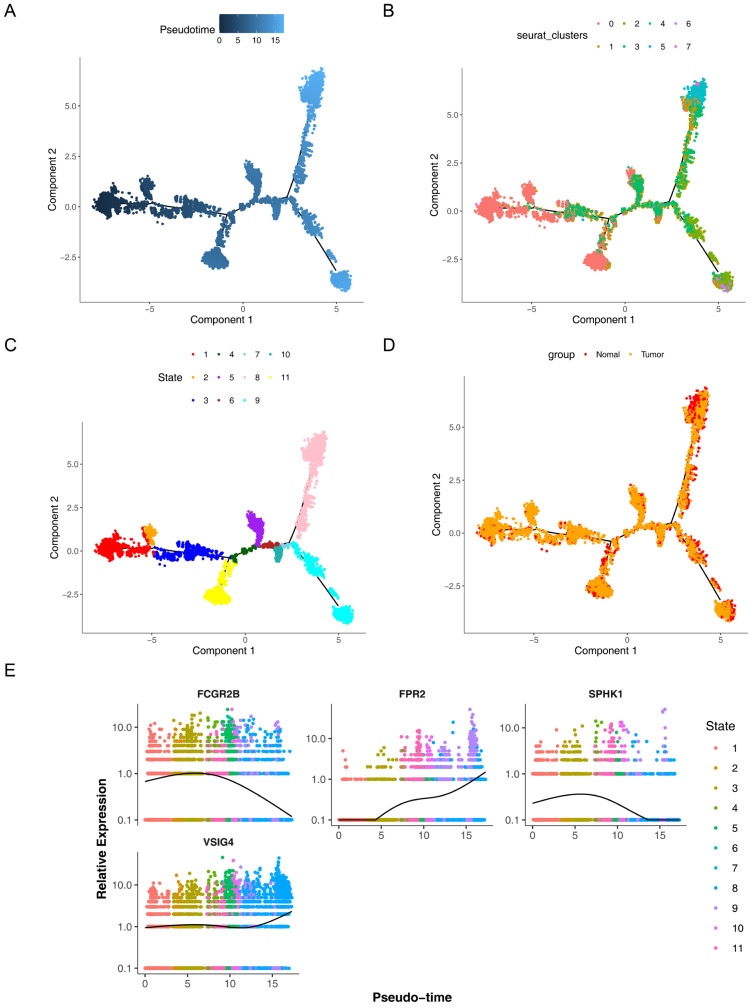
Pseudotemporal analysis of macrophage differentiation trajectories. (**A**) Pseudotemporal analysis diagram with darker shades representing early stages and lighter shades for later stages. (**B**,**C**) Macrophages categorized into eight distinct subgroups and eleven differentiation stages based on pseudotime ordering. (**D**) Distribution of macrophages across various differentiation stages in normal and tumor samples. (**E**) Relative expression of prognostic genes across different stages of macrophage differentiation.

Notably, significant differences in the abundance of these 10 cell types were observed between cancer and normal samples (*p* < 0.05). T cells and epithelial cells were more abundant in cancer samples, whereas NK cells and macrophages were less abundant in cancer samples compared to normal ones (Figure 8A). Differential expression of the four prognostic genes was observed between cancer and normal samples (*p* < 0.05) only in macrophages (Supplementary Figure S9), designating macrophages as crucial cells for subsequent analyses. The results showed that FCGR2B and VSIG4 expression in macrophages was upregulated in cancer samples, while FPR2 expression was downregulated. Further analyses verified that macrophages mainly interacted with fibroblasts via MIF-ACKR3 and HBEGF-EGFR, and exhibited enhanced interactions with epithelial and cancer cells through MIF-(CD74 + CD44) (Figure 8B,C, Supplementary Figure S10). Macrophages were then clustered into 8 subgroups, corresponding to 11 differentiation stages. Subgroup 0 was mainly detected in the predifferentiation stage, while subgroups 5 and 7 were mainly identified in the postdifferentiation stage (Figure 9A–D). Interestingly, the expression of SPHK1 and FCGR2B generally decreased during macrophage differentiation, whereas VSIG4 and FPR2 expression gradually increased (Figure 9E).

### 3.8. Spatial Validation of Macrophage Enrichment in CRC

**Figure 10:**
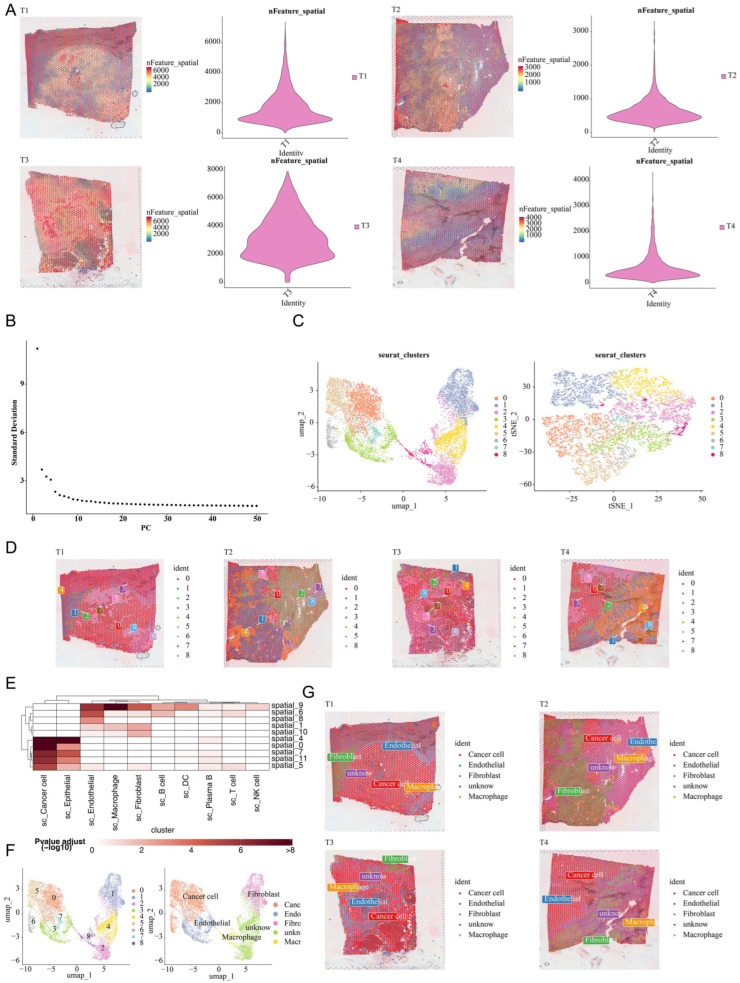
Spatial transcriptomics analysis validates macrophage enrichment in CRC tissues. (**A**) Quality control metrics showing nCount and nFeature distribution across four CRC samples. The left panels show the spatial distribution of gene expression counts (nCount) and the number of detected genes (nFeature) per spot, with warmer colors indicating higher values. The right panels show the overall distribution of nCount and nFeature across all spots. (**B**) ElbowPlot analysis used for optimal principal component selection, showing the standard deviation across different dimensions. (**C**) UMAP clustering plot displaying nine distinct spatial clusters identified in the integrated analysis. Each color represents a different cluster. (**D**) Spatial mapping of clusters onto individual CRC tissue sections, with numbers indicating cluster assignments. (**E**) Heatmap from multimodal intersection analysis (MIA) showing the enrichment of different cell types across spatial regions. The *X*-axis represents cell types identified from single-cell analysis, and the *Y*-axis represents spatial regions. Deeper colors indicate smaller *p*-values and stronger associations. (**F**) MIA-annotated UMAP plot shows the four identified major cell types: cancer cells, fibroblasts, macrophages, and endothelial cells. (**G**) Spatial mapping of MIA-identified cell types onto individual CRC tissue sections, with different colors representing different cell types.

Spatial transcriptomics analysis was performed to validate the spatial distribution patterns of macrophages in CRC tissues. Quality control analysis showed that CRC samples yielded an average of 3000 detected genes (nFeature) and 2000 total counts (nCount) per spot (Figure 10A). ElbowPlot analysis was used to determine the optimal number of principal components for downstream analysis (Figure 10B). Analysis of four CRC samples identified nine distinct spatial clusters using UMAP (Figure 10C,D). Multimodal intersection analysis (MIA) identified four major cell types with significant spatial enrichment: cancer cells, fibroblasts, macrophages, and endothelial cells (Figure 10E–G). Notably, MIA revealed that macrophages and epithelial cells were present at increased proportions in disease samples, consistent with our computational predictions based on macrophage-related phagocytosis regulatory genes.

### 3.9. Validation of the Expression of the Four Prognostic Genes in CRC

**Figure 11:**
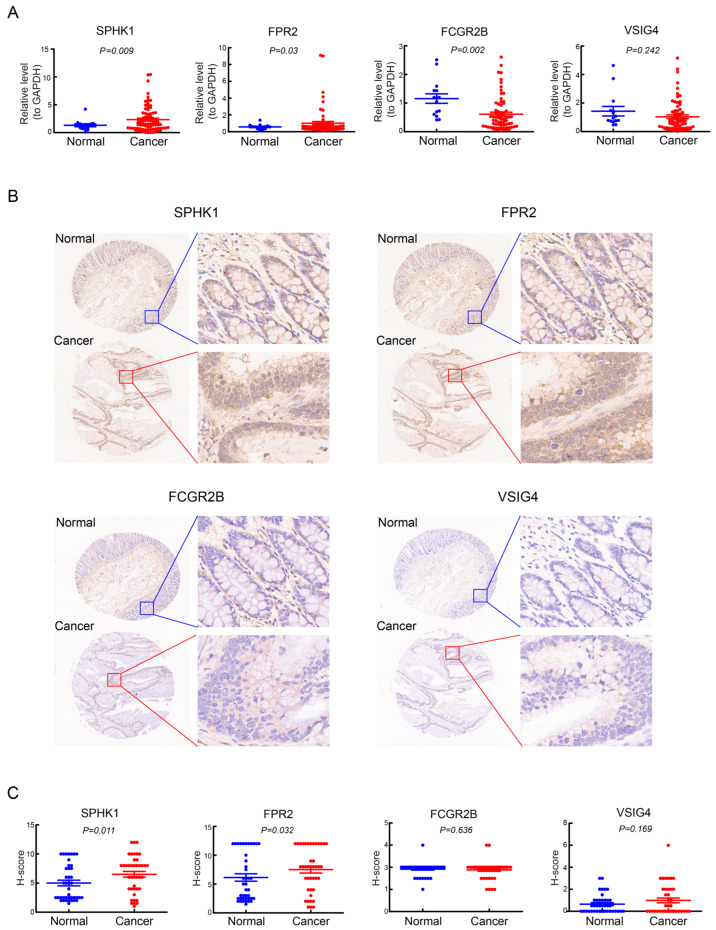
Validation of the four prognostic genes using qRT-PCR and IHC. (**A**) Expression levels of SPHK1, FPR2, FCGR1B and VSIG4 mRNAs in CRC and paired adjacent normal tissues were detected by qRT-PCR. (**B**) Representative images of immunohistochemical staining for SPHK1, FPR2, FCGR1B and VSIG4 in cancer and corresponding normal tissues. (**C**) Box plots showing the staining H-scores of both cancer tissues and paired normal tissues on tissue arrays.

The mRNA expression levels of the four prognostic genes were detected using human CRC cDNA microarrays (Figure 11A). The qRT-PCR data revealed significantly higher expression levels of SPHK1 (*p* < 0.01) and FPR2 (*p* < 0.05) mRNAs in CRC tissues compared to normal tissues, whereas the expression levels of FCGR2B mRNA were obviously lower in cancer tissues (*p* < 0.01). The expression of VSIG4 mRNA was slightly down-regulated, although this change was not statistically significant. To further verify the protein expression levels of the four genes in CRC, IHC analysis was performed on paraffin-embedded human CRC tissue microarrays (Figure 11B). The results indicated that the protein levels of SPHK1 (*p* < 0.05) and FPR2 (*p* < 0.05) were significantly upregulated in CRC tissues compared to adjacent normal tissues, while no significant differences were found in VSIG4 and FCGR2B expression (Figure 11C). Since VSIG4 is expressed exclusively in tissue macrophages and FCGR2B is restricted to B cells, the IHC method is not sufficiently sensitive for quantitative detection of these proteins in tissue samples.

### 3.10. Knockdown of SPHK1 Promotes M1 Polarization of Macrophages

**Figure 12:**
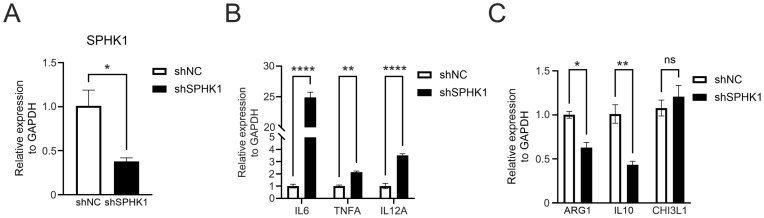
SPHK1 knockdown in SW480 cells modulates macrophage polarization in a co-culture system. (**A**) SW480 cells were infected with lentivirus encoding either control shRNA (shNC) or shRNA targeting SPHK1 (shSPHK1). Knockdown efficiency was confirmed by qRT-PCR. (**B**,**C**) PMA-induced macrophages were co-cultured with the aforementioned SW480 cells for 48 h. The mRNA levels of (**B**) M1 and (**C**) M2 macrophage marker genes in the macrophages were detected by qRT-PCR. * *p* < 0.05, ** *p* < 0.01, **** *p* < 0.0001, ns, not significant.

To identify the effect of SPHK1 on macrophage polarization, a co-culture system was established using CRC cells and PMA-activated THP-1 monocytes. THP-1 monocytes were first differentiated into M0 macrophages by treatment with 100 ng/mL PMA for 24 h in the lower chamber. SPHK1 knockdown SW480 cells were cultured in the upper chamber. A scramble shRNA served as the negative control (Figure 12A). The results demonstrated that upon co-culture with SPHK1 knockdown SW480 cells, PMA-activated THP-1 macrophages exhibited significantly upregulated expression of classic M1 markers, including IL6, TNFA, and IL12A. Conversely, the expression of M2-associated markers, Arg1 and IL10, was downregulated (Figure 12B,C). These findings suggest that SPHK1 knockdown in CRC cells promotes a shift in macrophage polarization toward the M1 phenotype, implying a potential role for SPHK1 in modulating the tumor immune microenvironment.

## 4. Discussion

Currently, surgery, chemotherapy and radiotherapy remain the primary methods for CRC, but the side effects associated with these strategies pose significant challenges [1]. In recent years, there has been growing interest in alternative treatments, such as immunotherapy, which has shown promising clinical outcomes. Given that CRC is a malignant cancer with high morbidity and mortality, the development of an accurate and effective CRC-related prognostic model is urgently needed.

TAMs are key components of the TME and play critical roles in tumorigenesis and progression [6]. TAMs are widely present in various cancers [7], and mounting evidence has linked the dysfunction of TAMs, including impaired phagocytosis, to the development and progression of CRC [19,36,37]. These findings suggest that macrophage-related phagocytic factors may be closely associated with CRC progression. Identifying biomarkers related to macrophage phagocytosis could provide valuable insights into the molecular mechanisms underlying CRC progression and potential therapeutic targets. In the present study, four phagocytosis-related genes (SPHK1, VSIG4, FCGR2B and FPR2) associated with CRC prognosis were identified. These results highlight the potential of these genes as prognostic markers for CRC. According to our findings, SPHK1 (sphingosine kinase 1), VSIG4 (V-set and immunoglobulin domain-containing 4), and FCGR2B (Fc gamma receptor IIb) were highly expressed in patients with higher risk scores, while FPR2 (formyl peptide receptor 2) was predominantly expressed in low-risk patients with CRC, aligning with previous studies. Numerous studies have confirmed that SPHK1 promotes invasion and metastasis in CRC [38,39]. High SPHK1 expression correlates with aggressive CRC behavior and poor OS, indicating that SPHK1 could serve as a potential prognostic biomarker and therapeutic target for patients with CRC [40]. VSIG4, a transmembrane protein specifically expressed on tissue-resident macrophages, plays a key regulatory role in tumor immunology [41,42]. VSIG4 is upregulated in multiple types of cancer, such as hepatocellular carcinoma, lung cancer, glioblastoma, and testicular cancer and its high expression is associated with poor prognosis [43,44,45]. Mechanistically, VSIG1 maintains an immunosuppressive tumor environment through inhibiting the activation of CD8+ T cells and IL-11 secretion and other alternative mechanisms [43,44]. Therefore, VSIG4 has been classified as a druggable gene according to the Drug-Gene Interaction database (DGIdb 3.0), which aggregates druggable gene categories and predicts druggability from multiple sources [46]. Our analysis of chemotherapy sensitivity in the two risk subgroups using a prognostic model with macrophage phagocytosis-related genes indicated that low-risk patients showed a greater response to immunotherapy, suggesting that VSIG4 may be a promising target for CRC therapy. FPR2, a seven-transmembrane G protein-coupled receptor, plays a crucial role in bacterial sensing and immune response modulation [47]. Furthermore, inhibition of aspirin-triggered specialized proresolving mediator (AT-SPM) biosynthesis or knockout of the AT-SPM receptor Alx/Fpr2 counteracted the immunomodulatory effects and diminished the protective impact of aspirin against CRC [48]. This finding supports the validity of our model, highlighting its relevance in CRC patients. FCGR2B is widely expressed on B cells, dendritic cells, various and myeloid cells (e.g., neutrophils, monocytes, and macrophages). It exhibits a context-dependent dual role in tumor immunity. On one hand, it suppresses B cell and other effector cell activation and reduces antibody production, thus preventing aberrant antibody response, inflammation and autoimmunity. On the other hand, recent studies also showed that it exerts some activating roles, for example, promoting cellular activation, cytotoxicity and phagocytosis, especially in monoclonal antibody immunotherapy [49,50,51,52]. At present, the role of FCGR2B in CRC remains unclear, but it has been implicated in the progression and phagocytosis of several other cancers [51,52,53]. Further investigation is needed to determine whether FCGR2B regulates CRC progression.

The TME plays a pivotal role in tumor initiation, progression, metastasis, and response to therapies. It consists of tumor cells interacting with surrounding immune cells, including macrophages, T cells, and NK cells [1,2,3]. Using a prognostic model based on macrophage phagocytosis-related genes across two risk subgroups, 14 critical TME cell types were identified: activated B cells, type 1 T helper cells, central memory CD8 T cells, MDSCs, effector memory CD8 T cells, neutrophils, activated CD4 T cells, T follicular helper cells, activated CD8 T cells, CD56dim NK cells, NK T cells, immature B cells, endothelial cells, and type 17 T helper cells. These findings underscore the critical role of these cells in CRC progression. These immune cells play key regulatory functions in finetuning the TME, influencing both the efficacy of immunotherapy and patient prognosis. Additionally, single-gene GSEA revealed that Th17 cell differentiation, type I diabetes mellitus, osteoclast differentiation, and the Toll-like receptor signaling pathway were linked to the macrophage phagocytosis-related genes selected in our model. These findings align with previous studies, indicating the involvement of these pathways in CRC tumorigenesis and progression [54,55,56,57].

Immune checkpoint therapy has gained significant attention and revolutionized tumor treatment in recent years. However, the efficacy of immunotherapeutics is often hindered by the presence of TAMs. TAMs are generally classified into two subtypes: M1 macrophages, which have tumor suppressive roles, and M2 macrophages, which promote tumor progression and inhibit immune responses [4]. M2 macrophages secrete a variety of molecules, including growth factors, metabolic products, cytokines, and chemokines, which block the activation of T and NK cells, thereby fostering tumor growth and immune suppression [4,5,6,7]. Recent studies have shown that VSIG4 promotes M2 polarization of macrophages and enhances CRC progression via HBEGF [58]. Our in vitro analyses demonstrated that knockdown of SPHK1 in SW480 cells promoted macrophage polarization toward M1 phenotype while inhibiting differentiation toward M2 phenotype. These findings suggest that our prognostic model can accurately predict immune cell subsets and phenotypes in the TME, as well as chemotherapy sensitivity in patients with CRC, offering valuable guidance for the selection of therapeutic strategies.

The clinical significance of the 14 TME cell types extends beyond prognostic evaluation to include therapeutic targeting and biomarker applications. Within adaptive immune cells, activated CD8+ T cells, effector memory CD8+ T cells, and central memory CD8+ T cells serve as key predictive biomarkers for response to PD-1/PD-L1 inhibitors, demonstrating significant overlap with established T-cell inflammatory signatures [59]. Type 1 T helper cells promote anti-tumor immunity through IFN-γ secretion and function as biomarkers for assessing inflammatory microenvironments, whereas Th17 cells exhibit a dual role in colorectal cancer, with their ratio serving as a potential biomarker for guiding IL-17 axis-targeted therapies [60]. Among immunosuppressive cell populations, myeloid-derived suppressor cells (MDSCs) represent promising therapeutic targets due to their potent immunosuppressive activity, which can be targeted using CXCR2 inhibitors, gemcitabine-mediated depletion, or ATRA-induced differentiation [61,62]. Neutrophils can polarize into N1 (anti-tumor) or N2 (pro-tumor) phenotypes, and their ratio may serve as a prognostic biomarker for predicting responses to anti-inflammatory therapies [63]. CD56dim NK cells and NKT cells hold significant potential in adoptive cell transfer and CAR-NK cell therapies, with recent clinical trials demonstrating efficacy in metastatic colorectal cancer patients [64,65]. Activated B cells contribute to antibody-dependent cellular cytotoxicity and may predict responses to monoclonal antibody therapies, while endothelial cells can serve as predictive biomarkers for anti-angiogenic treatment efficacy [66,67]. These cell types show considerable overlap with established immune markers, including classical Immunoscore signatures and ESTIMATE immune scores, and correspond to immune-inflamed, immune-excluded, and immune-desert cancer phenotypes [68,69]. This comprehensive cellular characterization provides a framework for developing immune stratification systems and guiding personalized immunotherapy choices for colorectal cancer patients.

Drug sensitivity analysis identified multiple compounds significantly associated with the risk score, several of which have been previously validated in CRC research, supporting the clinical relevance of our predictions. Irinotecan, a standard chemotherapeutic agent in CRC, demonstrated a negative correlation with the risk score in our analysis [70]. Dabrafenib has shown notable efficacy in BRAF-mutant CRC patients [71], and our predictive results indicate that high-risk patients may exhibit increased sensitivity to this drug. Furthermore, parthenolide and gambogic acid have been confirmed to exert anti-proliferative effects on CRC cells in multiple in vitro studies [72,73], consistent with our computational predictions. Although ponatinib is primarily used in other cancer types, it has also demonstrated anti-tumor activity in CRC cell lines [74]. The drug sensitivity predictions are based on well-validated computational methodologies: the TIDE (Tumor Immune Dysfunction and Exclusion) framework predicts immunotherapy response by evaluating features of T-cell dysfunction and exclusion [75], while the oncoPredict package employs machine learning models trained on large-scale pharmacogenomic databases (CTRP and PRISM) to predict individual patient drug responses based on tumor transcriptomic profiles [31]. This molecular feature-based approach to drug sensitivity prediction provides a robust theoretical foundation for personalized therapy in CRC.

The dynamic expression patterns of our prognostic genes during macrophage differentiation offer critical insights for therapeutic intervention strategies. Pseudotime analysis revealed that SPHK1 and FCGR2B expressions gradually decrease during macrophage differentiation, whereas VSIG4 and FPR2 expressions progressively increase, suggesting distinct therapeutic targeting opportunities at different stages of macrophage maturation. As a key enzyme in sphingolipid metabolism, SPHK1 represents a highly attractive therapeutic target, and several inhibitors are currently under investigation. Fingolimod (FTY720), an FDA-approved modulator of SPHK1, has demonstrated potential in cancer therapy by promoting anti-tumor immunity and reducing tumor growth [76]. Furthermore, specific SPHK1 inhibitors such as PF-543 and SKI-II have shown efficacy in preclinical cancer models by modulating macrophage polarization and enhancing immune surveillance [77]. Given its role in inflammation resolution and immune response regulation, FPR2 offers opportunities for both agonistic and antagonistic approaches. FPR2 agonists, including Resolvin D1 and Lipoxin A4, have demonstrated the ability to promote repolarization of M2 to M1 macrophages and enhance anti-tumor immunity [78]. Conversely, FPR2 antagonists may be beneficial in contexts where excessive inflammatory responses drive tumor progression. VSIG4, a negative regulator of T-cell activation, represents a promising target for combination immunotherapy. Although specific VSIG4 inhibitors are still in early development, targeting this pathway may enhance the efficacy of existing checkpoint inhibitors by alleviating macrophage-mediated immunosuppression [79]. Targeting FCGR2B provides another therapeutic avenue. Anti-FCGR2B antibodies are currently being explored for their ability to enhance antibody-dependent cellular cytotoxicity and improve the efficacy of therapeutic antibodies [80]. The temporal expression dynamics we observed suggest that targeting these genes at specific stages of macrophage differentiation may maximize therapeutic benefits while minimizing off-target effects. Future treatment strategies should consider integrating these targets with existing immunotherapies to achieve synergistic anti-tumor effects in CRC patients.

This study successfully established a four-gene prognostic model based on macrophage-related phagocytosis regulatory genes through the integration of bioinformatic analysis, single-cell sequencing, and experimental validation. The model provides novel molecular markers for risk stratification, prediction of immunotherapy sensitivity, and evaluation of chemotherapy response in colorectal cancer patients. It also reveals the dynamic expression patterns of these regulatory factors during macrophage differentiation and their crucial roles in remodeling the tumor microenvironment. However, several limitations of this study should be noted. First, the sample size of the validation cohort was relatively small, and the training and validation sets were derived from different sequencing platforms. Such technical discrepancies may affect the generalizability and extrapolation capacity of the model. Second, the current conclusions are largely based on bioinformatic associations and computational predictions. The model has yet to be prospectively validated in real-world clinical cohorts, and systematic in vitro experimental evidence is still insufficient, making it difficult to establish definitive causal relationships. Furthermore, the predictive performance of the model (e.g., AUC values at a moderate level) still requires improvement, and its association with established therapeutic targets (e.g., EGFR/VEGF) remains insufficiently explored. To address these limitations, future studies should focus on the following aspects: First, prospective multi-center clinical studies with larger sample sizes, particularly including cohorts of CRC patients receiving immunotherapy, should be conducted to validate the clinical utility of the model. Second, well-designed in vitro functional experiments are needed to elucidate the molecular mechanisms through which these genes regulate macrophage function and chemotherapy sensitivity. Third, a systematic investigation into the synergistic effects between the model and existing therapeutic targets should be carried out to assess its potential for clinical translation. Finally, the incorporation of advanced computational approaches, such as multi-omics integration and machine learning optimization, along with standardized batch effect correction methods (e.g., ComBat algorithm), will further enhance the predictive accuracy and cross-platform applicability of the model.

## 5. Conclusions

This study presents a four-gene (SPHK1, VSIG4, FCGR2B, and FPR2) signature to predict the prognosis of patients with CRC. The results confirmed that this four-gene prognostic signature, associated with phagocytosis-related genes, demonstrates strong predictive performance and serves as an independent prognostic factor. Furthermore, the investigation of the TME and chemotherapy sensitivity in CRC may provide clinical insights for future treatment strategies. This is the first report of a macrophage phagocytosis-related gene signature for prognostic prediction in CRC. Despite the model’s current limitations, further experiments and validation across large cohorts will optimize and refine this prognostic model, enhancing its accuracy and clinical applicability.

## Data Availability

The datasets analyzed in the current study are available in The Cancer Genome Atlas (TCGA) database (https://www.cancer.gov/tcga/ (accessed on 30 December 2022)) and the Gene Expression Omnibus (GEO) database (http://www.ncbi.nlm.nih.gov/geo/ (accessed on 30 December 2022)).

## Supplementary Materials

The following supporting information can be downloaded at: https://www.mdpi.com/article/10.3390/cimb47100804/s1.

## Abbreviations

The following abbreviations are used in this manuscript:

CRC	colorectal cancer
TCGA	The Cancer Genome Atlas
GEO	Gene Expression Omnibus
GSEA	gene set enrichment analysis
GO	Gene Ontology
KEGG	Kyoto Encyclopedia of Genes and Genomes
ssGSEA	single-sample GSEA
LASSO	least absolute shrinkage and selection operator
qRT-PCR	quantitative real-time polymerase chain reaction
IHC	immunohistochemistry
TME	tumor microenvironment
TAMs	tumor-associated macrophages
MMPs	matrix metalloproteinases
PD-L1	programmed cell death 1 ligand 1
scRNA-seq	single-cell RNA sequencing
TMB	tumor mutation burden
TIDE	Tumor Immune Dysfunction and Exclusion
UMI	unique molecular identifier
VST	variance stabilizing transformation
UMAP	uniform manifold approximation and projection
AUC	area under the curve
MIF	macrophage migration inhibitory factor
SPHK1	sphingosine kinase 1
VSIG4	v-set and immunoglobulin domain-containing 4
FCGR2B	Fc gamma receptor IIb
FPR2	formyl peptide receptor 2
AT-SPM	aspirin-triggered specialized proresolving mediators
