# Supplementary material for: Identification and Validation of a Macrophage Phagocytosis-Related Gene Signature for Prognostic Prediction in Colorectal Cancer (CRC)
Source: Curr Issues Mol Biol. 2025 Sep 29;47(10):804. doi: 10.3390/cimb47100804 (PMC12562277; doi:10.3390/cimb47100804)
Supplement: Supplementary file 1 [file cimb-47-00804-s001.zip › Supplementary Figures.pdf]

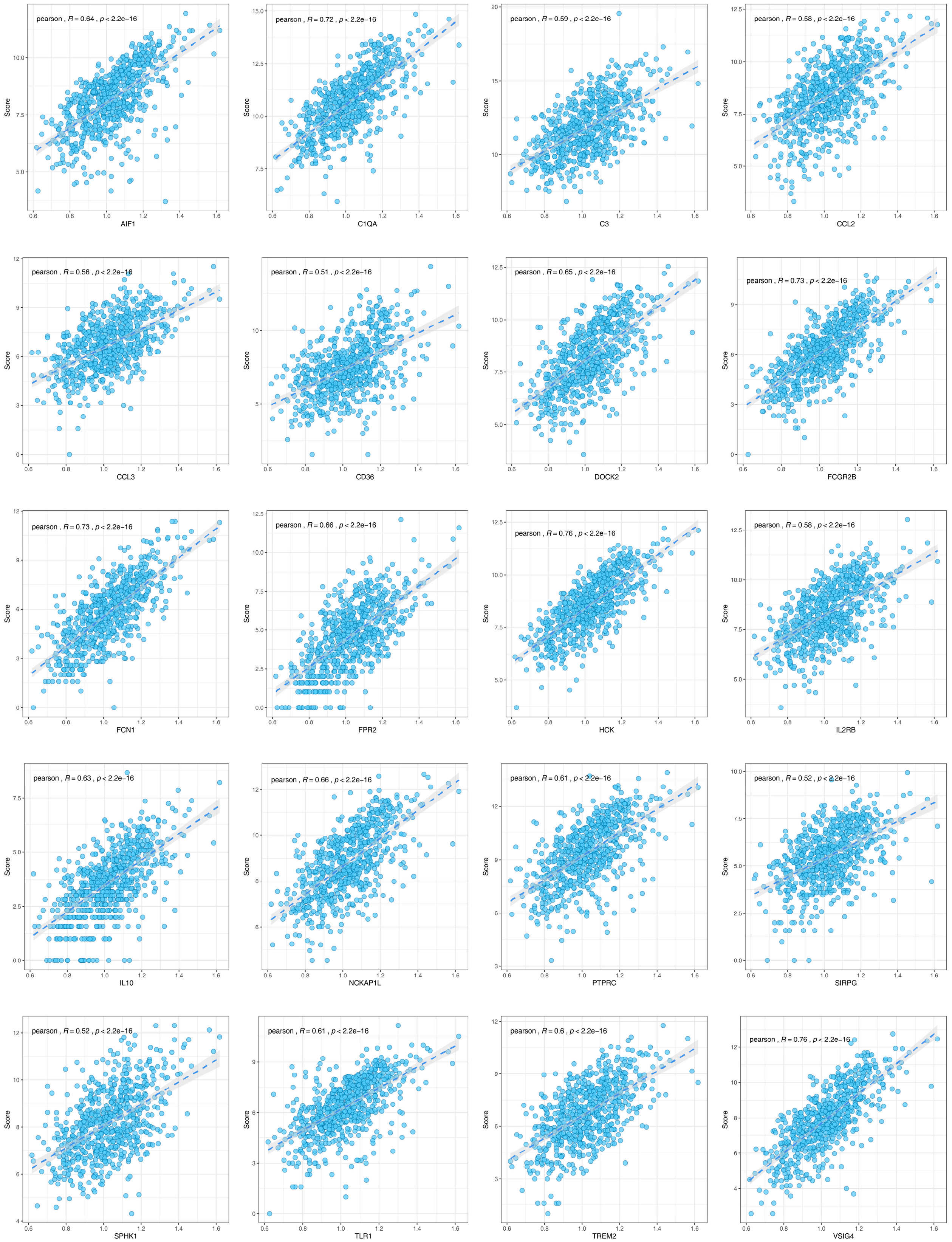

Supplementary Figure S1. Correlation analysis between the expression levels of 20 phagocytosis regulators and macrophage enrichment scores.

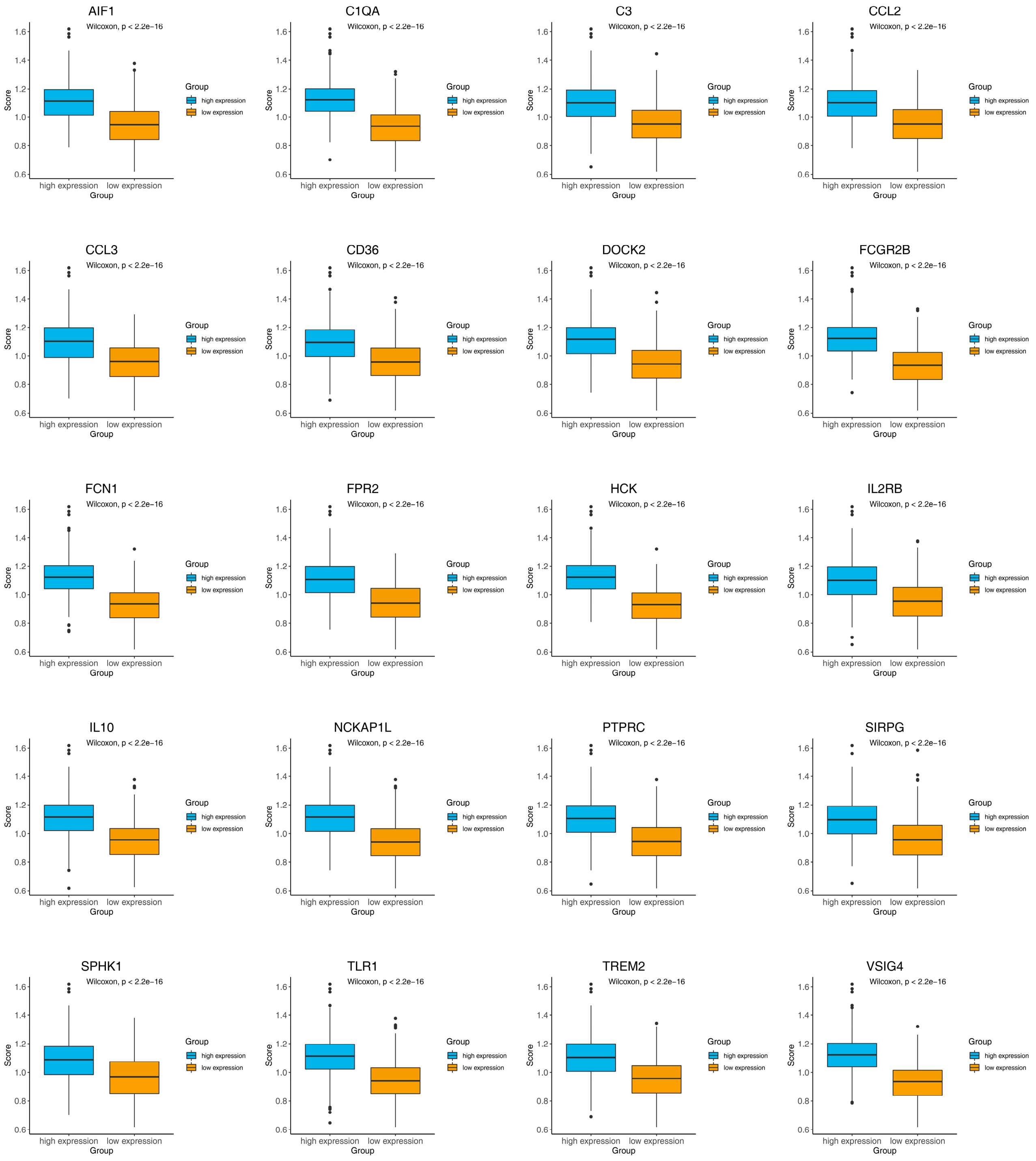

Supplementary Figure S2. Comparison of macrophage enrichment scores between high and low expression of phagocytosis regulators.

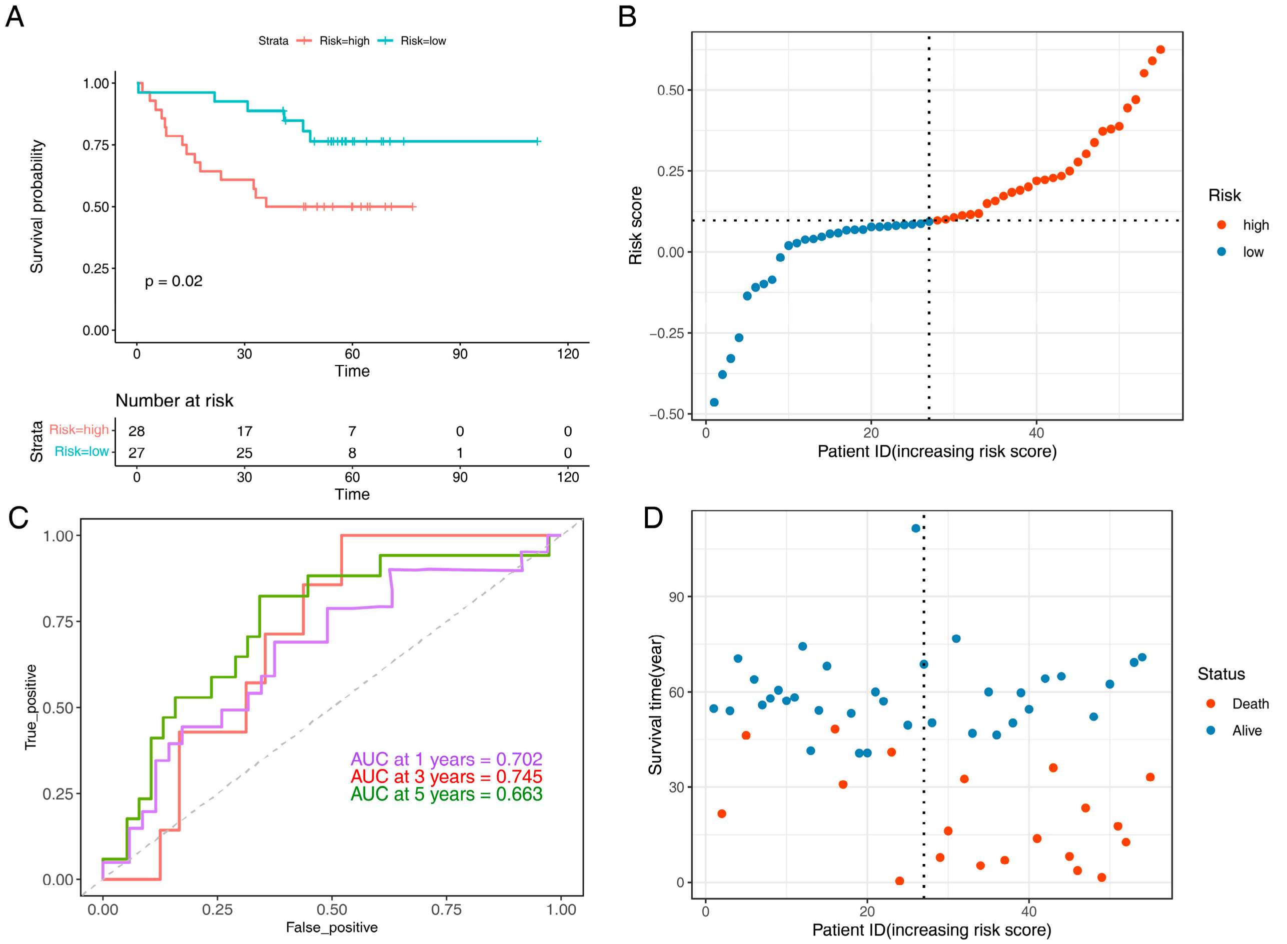

Supplementary Figure S3. Validation of the prognostic model using the GSE17537 dataset. (A) K-M curve indicates the significant difference in survival between the high- and low-risk groups. (B) The risk score curve classified CRC patients into high- and low-risk groups using an optimal threshold. (C) ROC curves confirmed the effectiveness of the prognostic model. (D) The scatter plot displays the survival status of patients in the GSE17537 dataset.

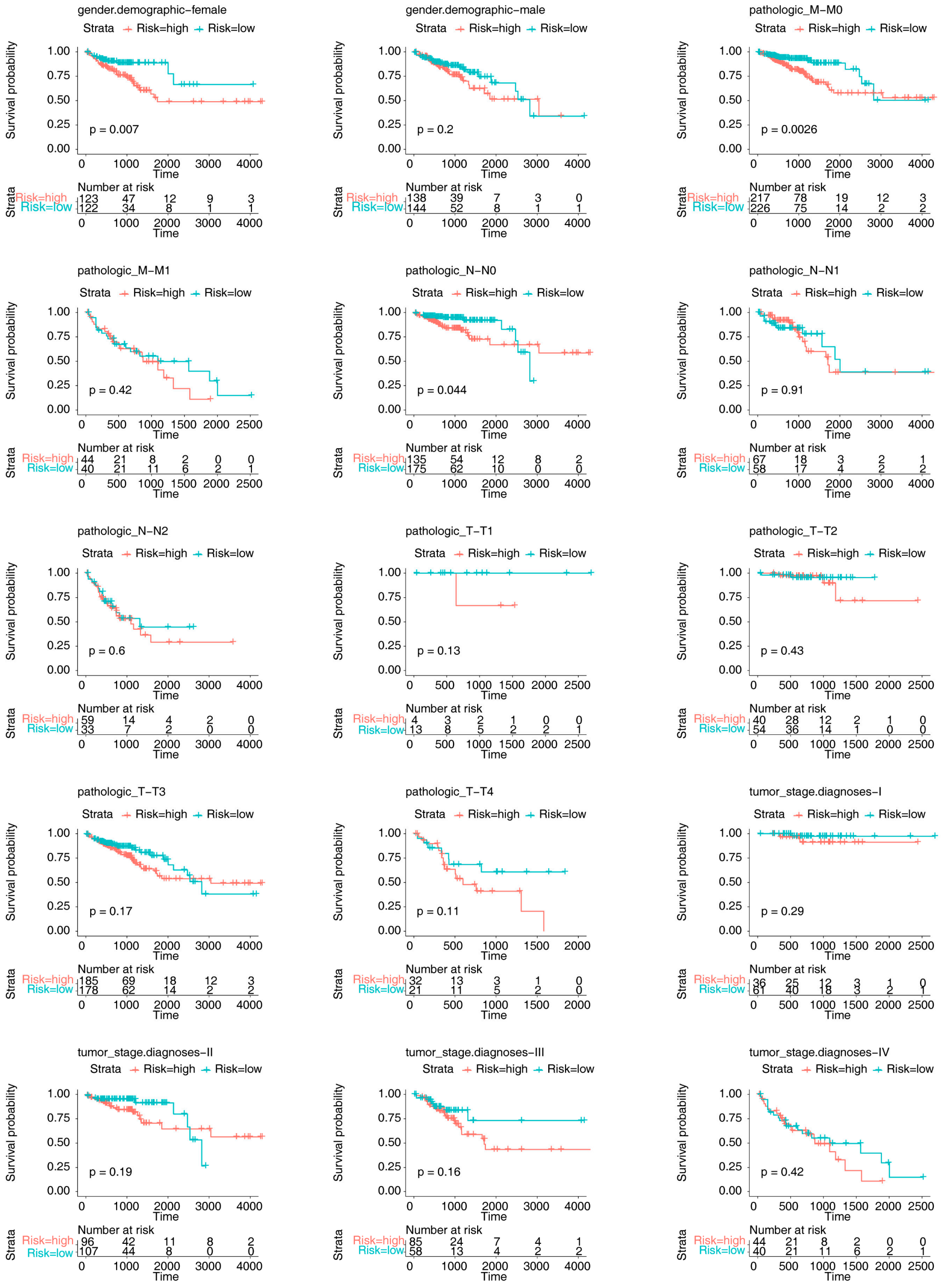

Supplementary Figure S4. K-M survival curves indicate the differences in subgroups divided by different clinical features.

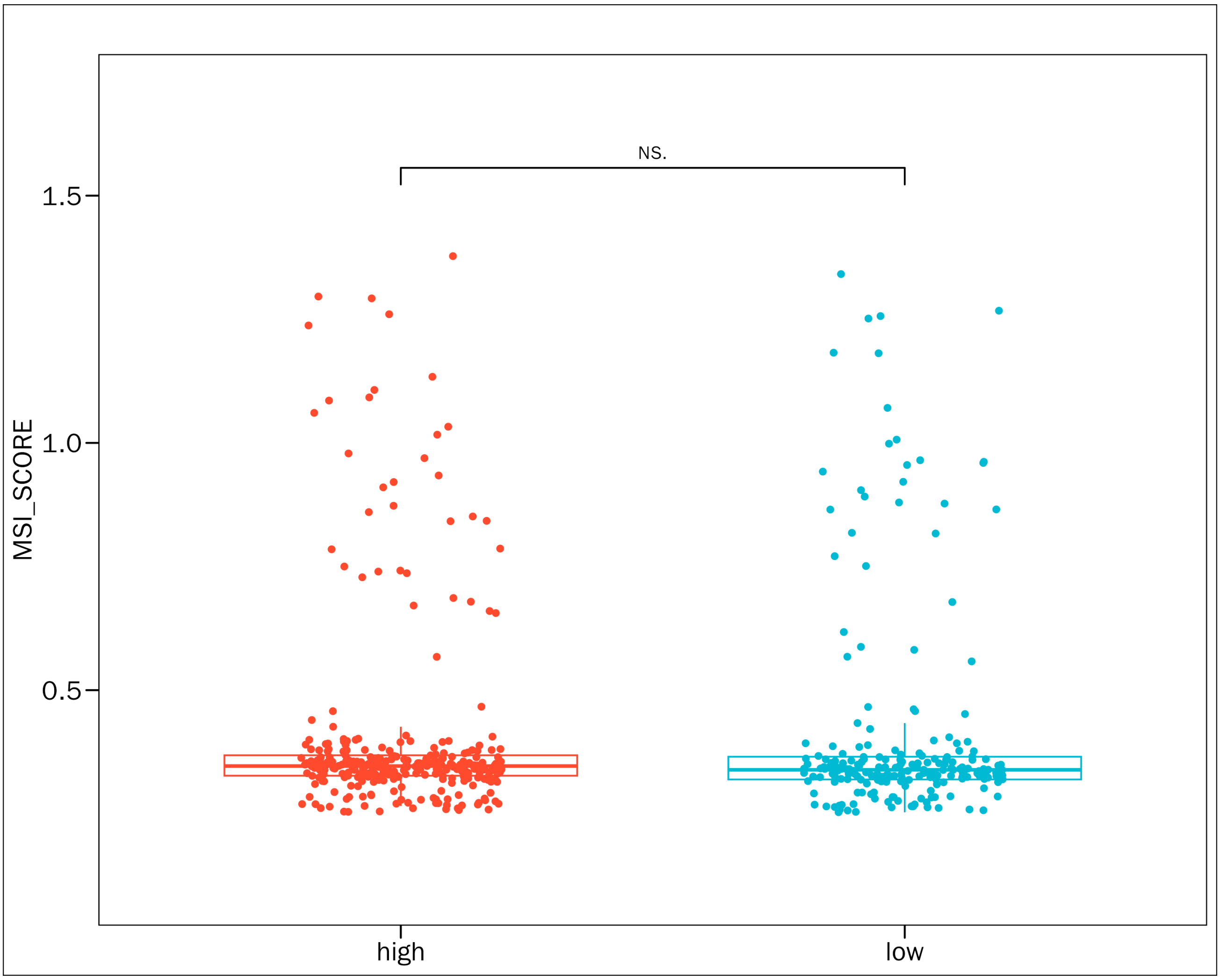

Supplementary Figure S5. Comparison of microsatellite instability (MSI) scores between the high- and low-risk groups.

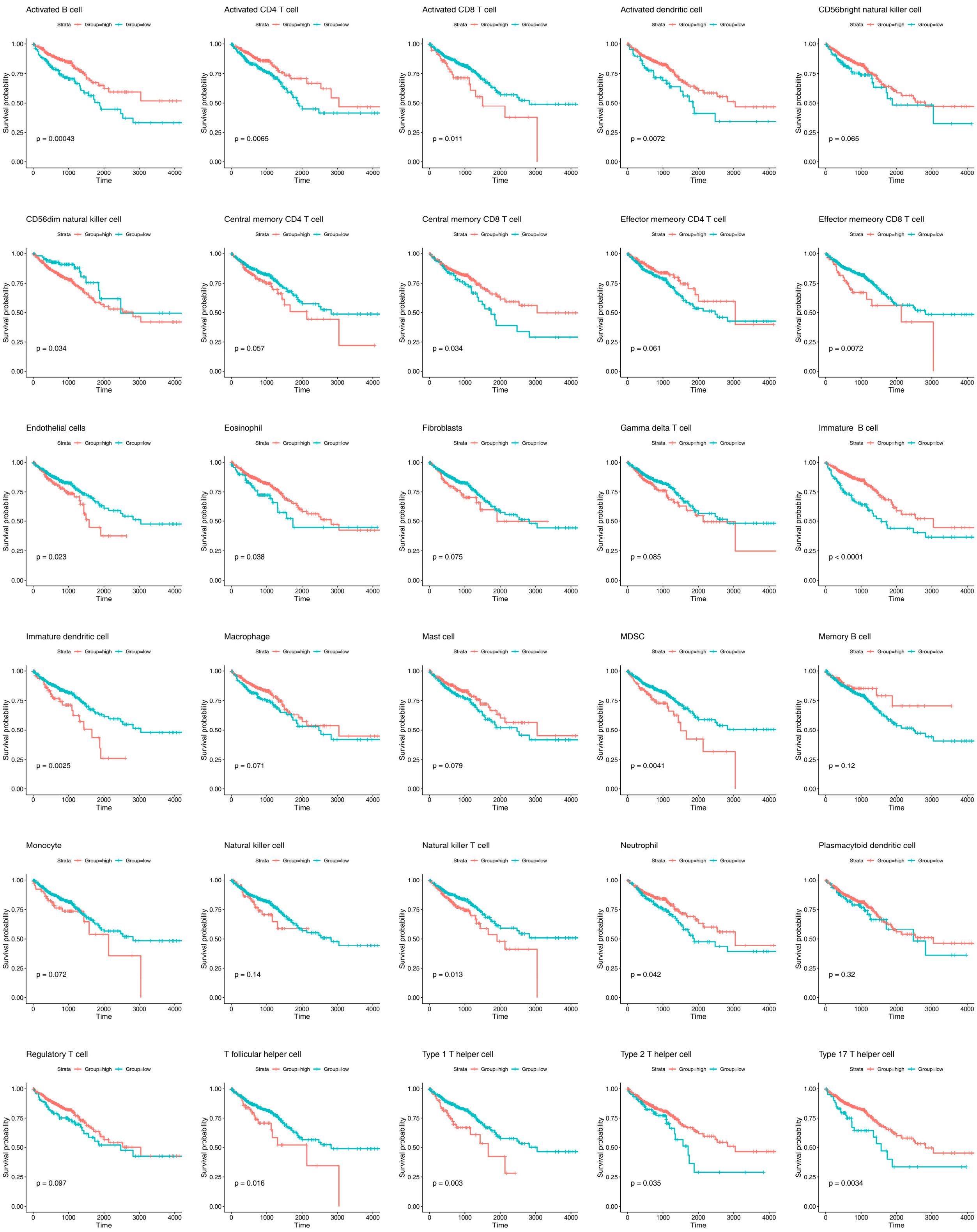

Supplementary Figure S6. K-M curves indicate the survival value of 18 types of TME cells in CRC.

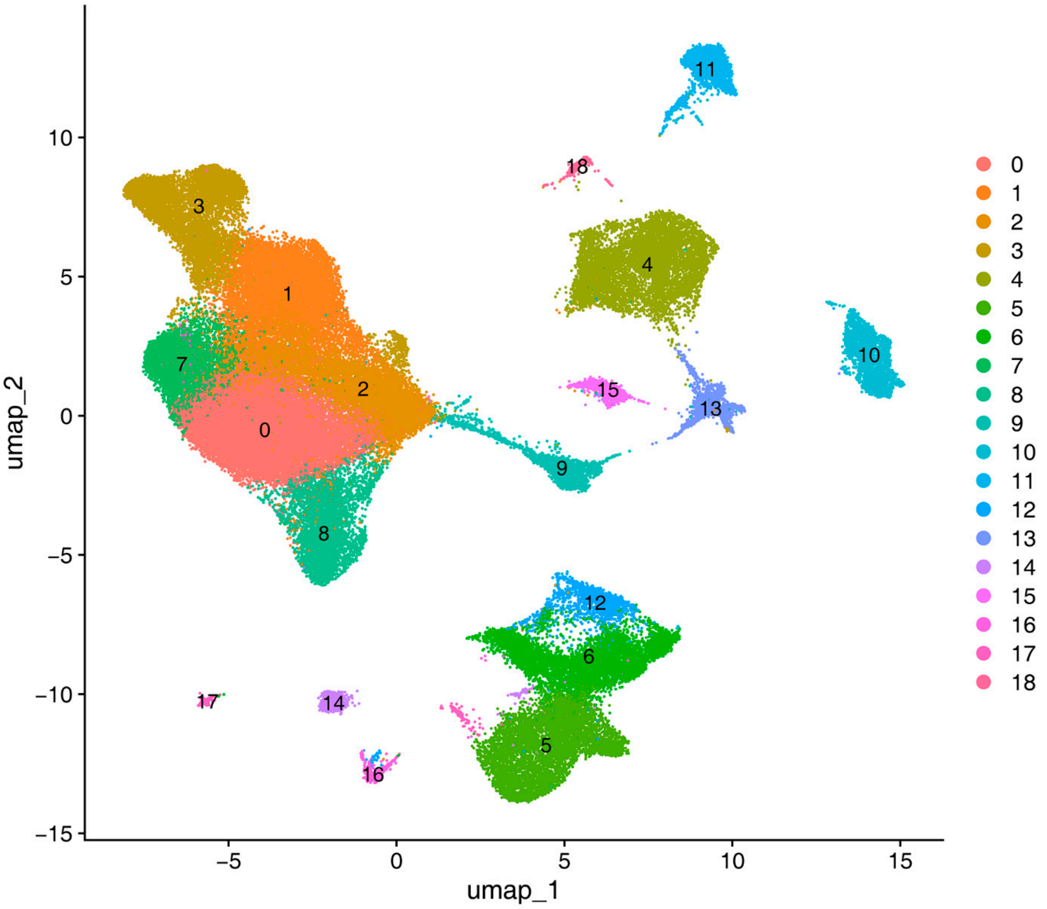

Supplementary Figure S7. UMAP plot of scRNA-seq data from the GSE231559 dataset showing 19 clusters.

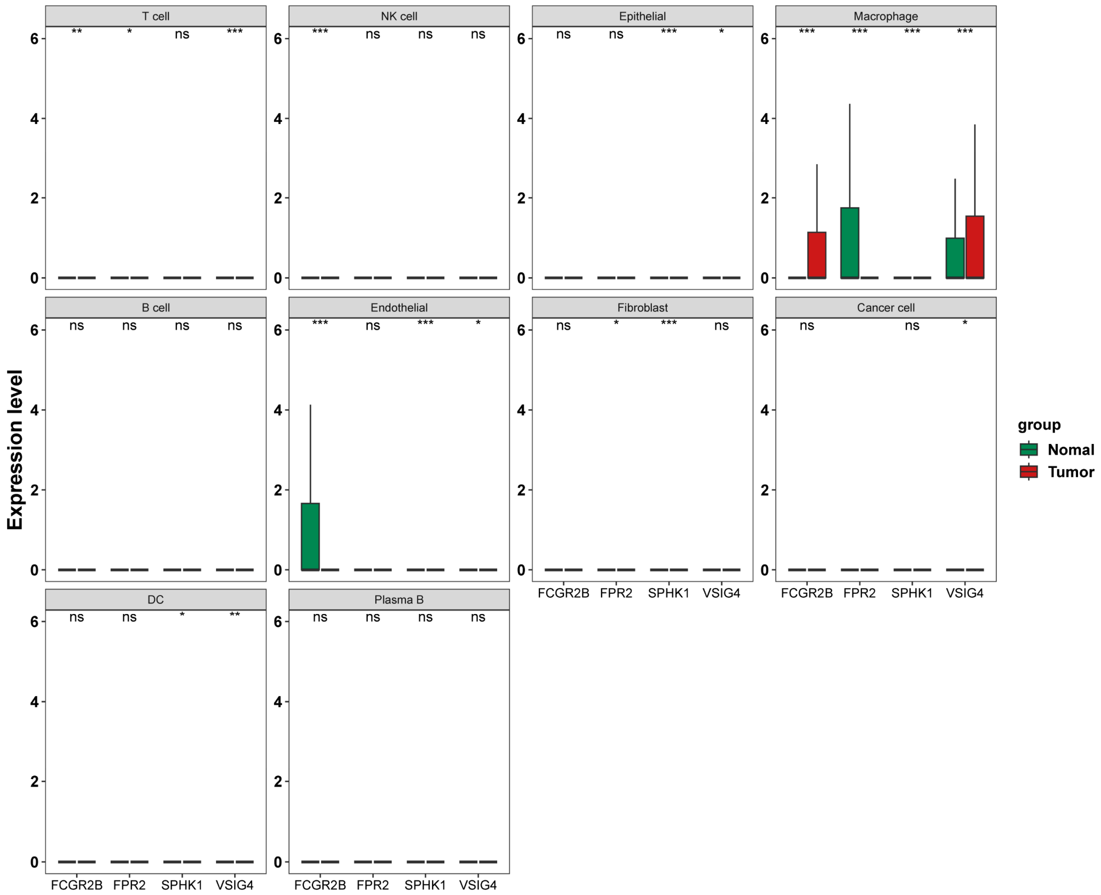

Supplementary Figure S8. The expression of prognostic genes in 10 cell types, comparing normal and tumor samples.

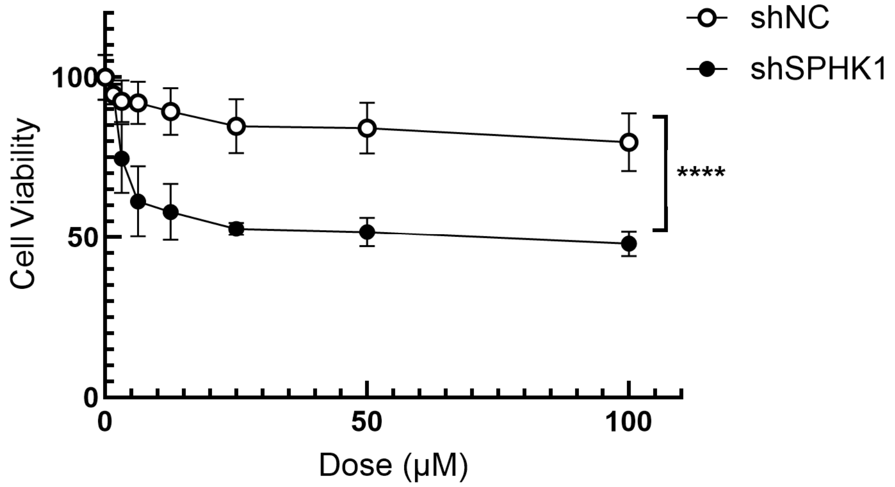

Supplementary Figure S9. Knockdown of SPHK1 sensitizes SW480 colorectal cancer cells to 5-fluorouracil (5-FU). SW480 cells were infected with lentivirus encoding either control shRNA (shCtrl) or shRNA targeting SPHK1 (shSPHK1). Dose–response curves of SW480 cells following treatment with 5-FU for 72 hours. Cell viability was normalized to untreated controls (0 μM 5-FU) and measured via CCK-8 assay. **** $P < 0.0001$.

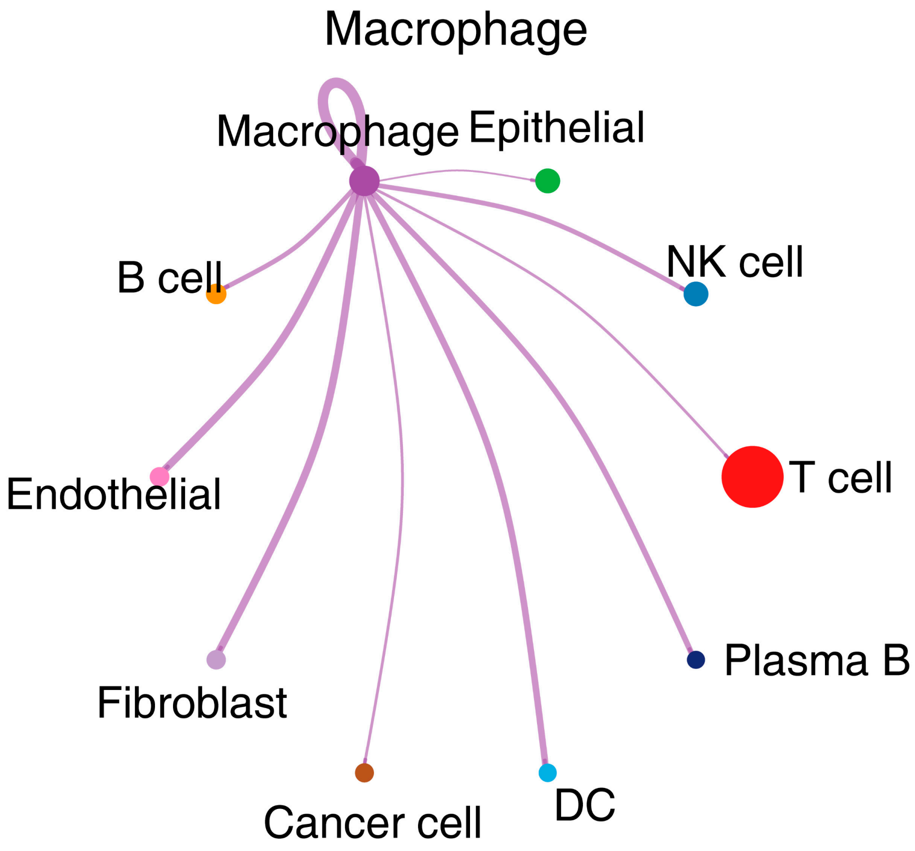

Supplementary Figure S10. A network diagram illustrating the interactions between macrophages and other cell types.
