# Supplementary material for: Identification and Validation of a Macrophage Phagocytosis-Related Gene Signature for Prognostic Prediction in Colorectal Cancer (CRC)
Source: Curr Issues Mol Biol. 2025 Sep 29;47(10):804. doi: 10.3390/cimb47100804 (PMC12562277; doi:10.3390/cimb47100804)
Supplement: Supplementary file 1 [file cimb-47-00804-s001.zip › Supplementary Table S1.pdf]

	level	TCGA-CRC	GSE17537	p
n		585	55	
Age (%)	<60	187 (32.0)	25 (45.5)	0.06
	>60	398 (68.0)	30 (54.5)	
Gender (%)	Female	270 (46.2)	29 (52.7)	0.428
	Male	315 (53.8)	26 (47.3)	
Stage (%)	I	106 (18.1)	4 (7.3)	0.003
	II	219 (37.4)	15 (27.3)	
	III	174 (29.7)	19 (34.5)	
	IV	86 (14.7)	17 (30.9)	
OS (%)	Alive	471 (80.5)	35 (63.6)	0.006
	Died	114 (19.5)	20 (36.4)	
OS.time (%)	< 1 year	123 (21.0)	10 (18.2)	<0.001
	1-3 years	314 (53.7)	23 (41.8)	
	3-5 years	100 (17.1)	7 (12.7)	
	> 5 years	48 (8.2)	15 (27.3)	
